# Chip-based label-free incoherent super-resolution optical microscopy

**DOI:** 10.1038/s41377-025-01914-x

**Published:** 2025-08-04

**Authors:** Nikhil Jayakumar, Luis E. Villegas-Hernández, Weisong Zhao, Hong Mao, Firehun T. Dullo, Jean-Claude Tinguely, Krizia Sagini, Alicia Llorente, Balpreet Singh Ahluwalia

**Affiliations:** 1 Department of Physics and Technology, UiT The Arctic University of Norway, Tromsø, 9037 Norway; 2 Innovation Photonics and Imaging Center, State Key Laboratory of Space Environment Interation with Matters, Key Laboratory of Ultra-Precision Intelligent Instrumentation of Ministry of Industry and Information Technology, School of Instrumentation Science and Engineering, Harbin Institute of Technology, Harbin, 150080 China; 3 SINTEF Digital department of Microsystems and Nanotechnology, Gaustadalleen 23C, 0373 Oslo, Norway; 4 Department of Molecular Cell Biology, Institute for Cancer Research, Oslo University Hospital, The Norwegian Radium Hospital, 0379 Oslo, Norway; 5 Centre for Cancer Cell Reprogramming, Faculty of Medicine, University of Oslo, Montebello, 0379 Oslo Norway; 6 Department for Mechanical, Electronics and Chemical Engineering, Oslo Metropolitan University, Oslo, Norway; 7 Department of Clinical Science, Intervention and Technology, Karolinska Institute, Solna, Sweden

**Keywords:** Super-resolution microscopy, Integrated optics

## Abstract

The photo-kinetics of fluorescent molecules have enabled the circumvention of the far-field optical diffraction limit. Despite its enormous potential, the necessity to label the sample may adversely influence the delicate biology under investigation. Thus, continued development efforts are needed to surpass the far-field label-free diffraction barrier. The statistical similarity or finite coherence of the scattered light off the sample in label-free mode hinders the application of existing super-resolution methods based on incoherent fluorescence imaging. In this article, we present physics and propose a methodology to circumvent this challenge by exploiting the photoluminescence (PL) of silicon nitride waveguides for near-field illumination of unlabeled samples. The technique is abbreviated EPSLON, Evanescently decaying Photoluminescence Scattering enables Label-free Optical Nanoscopy. We demonstrate that such an illumination has properties that mimic the photo-kinetics of nano-sized fluorescent molecules, i.e., such an illumination permits incoherence between the scattered fields from various locations on the sample plane. Thus, the illumination scheme enables the development of a far-field label-free incoherent imaging system that is linear in intensity and stable over time, thereby permitting the application of techniques like structured illumination microscopy (SIM) and intensity-fluctuation-based optical nanoscopy (IFON) in label-free mode to circumvent the diffraction limit. In this proof-of-concept work, we observed a two-point resolution of $\sim$180 nm on super-resolved nanobeads and resolution improvements between 1.9× to 2.8× over the diffraction limit, as quantified using Fourier Ring Correlation (FRC), on various biological samples. We believe EPSLON is a step forward within the field of incoherent far-field label-free super-resolution microscopy that holds a key to investigating biological systems in their natural state without the need for exogenous labels.

## Introduction

The ability of light beams to interfere is quantified by their degree of coherence. Light beams originating from within the coherence volumes can only overlap and generate a sustained interference pattern^1,2^. In fluorescence microscopy, the transversal coherence lengths are typically on the order of a few nanometers. This is because the fluorescent molecules, a few nanometers in size, emit independently and stochastically. This leads to a linear mapping between the sample plane fluorophore concentration and the image plane intensity. This may be utilized to circumvent the far-field diffraction-limit, as in fluorescence-based SIM^3,4^ or fluorescence-based IFON algorithms^5–10^. However, the absence of such exogenous molecules in label-free microscopy restricts the far-field transversal coherence lengths to a few hundred nanometers^11,12^. This hinders the application of fluorescence-based super-resolution algorithms in the label-free regime for generating reliable super-resolved images^13^. Another hindrance in label-free microscopy is the lack of selectivity and specificity that results in strong scattering and multiple scattering from the entire sample. To alleviate these scattering challenges, near-field illumination via nano-sized light sources with stochastic photo-kinetics and sufficient quantum yield will be beneficial. Thus, through this article, a concept is introduced to solve the challenge of generating far-field two-dimensional label-free super-resolved optical images using fluorescence-based super-resolution algorithms: photoluminescence (PL) of silicon nitride (Si_3_N_4_)^14,15^ waveguide functions as exogenous nano-sized illumination sources with stochastic photo-kinetics. That is, the evanescently decaying part of the intrinsic incoherent PL generated in Si_3_N_4_ gets converted into propagating waves upon interaction with the unlabeled sample placed on the core. Then the waveguide helps in engineering the illumination beam profile to induce fluctuations in intensity at the sample plane either via multi-mode interference (MMI)/speckle-like patterns^16–18^ or via well-defined interference fringes. This permits the application of fluorescence-based IFON algorithms^19,20^ or SIM^3,4^ respectively to enhance the resolution.

Two major impediments to the development of label-free optical microscopy are poor contrast and diffraction-limited resolution. To mitigate the issue of poor contrast, various approaches have emerged such as phase contrast microscopy^21^, differential interference contrast^22^, Hoffman modulation^23^, interferometric scattering microscopy^24^, quantitative phase microscopy^25^, holographic non-interferometric techniques^26^, Fourier Ptychography^27^, rotating coherent scattering microscopy^28^, manipulation of the coherence of light sources used for illumination^29^, ultraviolet microscopy^30^, optical waveguides^31,32^, gold nanoparticle tagging along with temporally sequenced labeling^33^, among others. However, circumventing the diffraction limit in the label-free regime is still challenging in life sciences, as opposed to fluorescence microscopy^34^. This could be attributed to the ease of utilizing/manipulating the photo-kinetics of nano-sized fluorescent molecules to gain information beyond the diffraction limit.

For label-free super-resolution microscopy, the different approaches developed include near-field scanning optical microscopy^35^, super-lens^36^, micro-sphere assisted super-resolution imaging^37^, high-index liquid immersed microsphere assisted super-resolution^38^, optical super-oscillation techniques^39^, non-linear imaging systems^40^, multiplexing information in polarization or wavelength^41^, multiplexing information in time via dynamic random flow of a sparse set of nanoparticles that help capture sub-diffraction sized sample features by converting evanescent waves into propagating waves^42^, utilizing intrinsic autofluorescence of biological specimens in tandem with fluorescence-based super-resolution algorithms^43,44^ and others. These approaches have their respective experimental challenges, especially for life sciences applications.

Different concepts have been developed to enhance the resolution and contrast of label-free optical techniques in the far-field^45^. But these coherent scattering methods are still limited by Abbe’s diffraction limit. A short overview of these approaches is included in Supplementary Section 1. Broadly, these methods employ the concept of synthetic aperture or spatial frequency shifting for coherently scattering specimens using free-space optics^27,28,46–48^ or chip-based solutions^31,49–51^, or the application of fluorescence-based super-resolution algorithms to coherently scattering specimens^52^. Some of these techniques achieve sub-100 nm resolution, but the best achievable theoretical resolution is still given by the Abbe-limit, $\frac{{\lambda }_{det }}{{{NA}}_{{ill}}+{{NA}}_{det }}$, where ${\lambda }_{\det }$ is the wavelength of the detected light and ${\rm{NA}}_{\rm{ill}/\det }$ is the numerical aperture of the illumination and detection light paths, respectively. Therefore, these techniques are diffraction-limited and not classified as super-resolution as they cannot achieve infinite resolution in principle.

**Fig. 1:**
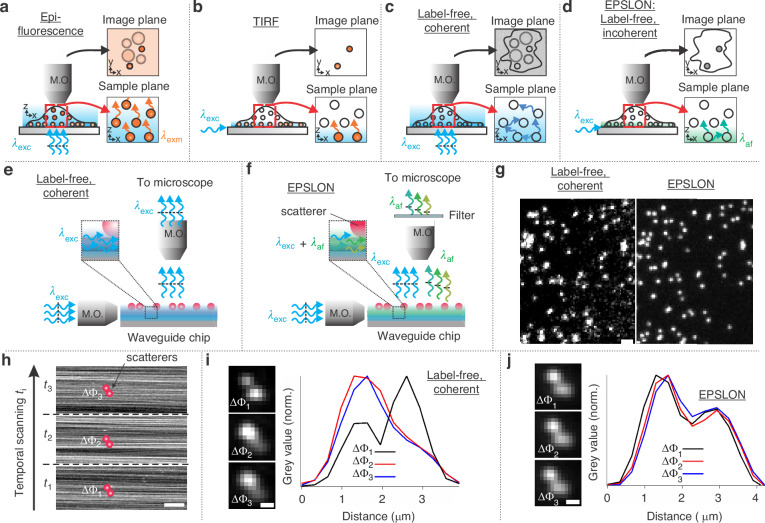
Overview of EPSLON. **a**–**d** Comparison between fluorescence and label-free microscopy. **a** Epifluorescence: coherent light *λ*_exc_ is used for excitation of fluorescent molecules, and the camera detects the Stokes-shifted incoherent light emitted by the molecules *λ*_em_. Stochastic fluctuations of the Stokes-shifted light and specificity offered by the molecules help suppress speckle noise, enabling high-contrast imaging. **b** TIRF: coherent light for near-field illumination of fluorescently labeled samples, and incoherent light gets detected by the camera. Near-field illumination helps to further improve the contrast as compared to the epifluorescence by illuminating thin sections of the sample. **c** Label-free coherent imaging: coherent light for illumination, and coherent light gets detected by the camera. Multiple scattering and the coherent nature of the scattered light lead to speckle noise, **d** EPSLON: incoherent light for near-field illumination of unlabeled samples and incoherent light scattered by the sample, *λ*_af_, forms the image. The incoherent nature of the detected light, in addition to the near-field illumination, helps generate high-contrast label-free images. **e** Schematic of optical waveguide-based label-free coherent imaging. The guided coherent light generates an evanescent field that interacts with the sample placed at the core-cladding interface. **f** Schematic of EPSLON imaging using Si_3_N_4_ waveguide. The guided coherent light induces incoherent photoluminescence (PL) in the core of the waveguide that interacts with the sample and gets transmitted into the far-field. **g** 200 nm gold nanoparticles imaged in coherent and EPSLON mode, scale bar 100 μm. The issues of coherent noise, poor-contrast associated with conventional label-free techniques is mitigated in EPSLON due to ${\rm{\delta }}$-function correlations existing in the detected light. **h** Temporal scanning of the coupling objective helps induce fluctuations in image intensity over time. To illustrate the principle, two particles, shown in red, are placed on top of a waveguide core. At each instance of time *t*_*i*_, different MMI patterns get generated along the length of the waveguide as showcased by the experimental images, scale bar 10 μm. The particles scatter the guided light into the far-field with different phases Δ*φ*_i_, and an image is acquired. **i** Experimental demonstration of coherence of scattered light using two 200 nm gold nanoparticles, scale bar 10 μm. The excitation of different modes causes the phase difference Δ*φ*_i_ between the scattered light off the particles to change, leading to different images at different instances of time *t*_*i*_, in label-free coherent imaging. This is demonstrated by the graphs provided alongside, which showcase the intensity variation along the respective colored lines in the images of the two particles provided. **j** Experimental realization of the stochastic nature of the detected light in label-free imaging. The same nanoparticles shown in **i** are imaged in EPSLON mode, scale bar 10 μm. In EPSLON, stochastic fluctuations between the scattered incoherent PL light reaching the camera lead to identical images at different instances of time *t*_*i*_. This can be seen from the graphs showcasing intensity variations along the respective colored lines in the bead images provided alongside

**Fig. 2:**
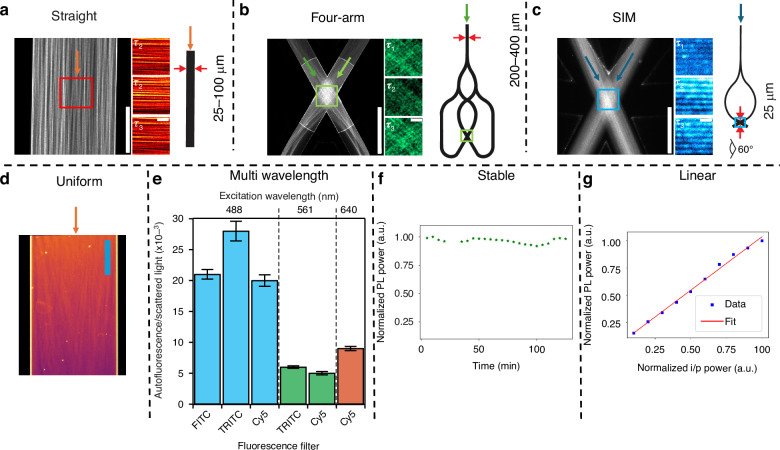
Structuring the incoherent photoluminescent illumination using Si_3_N_4_ waveguides and its properties. **a**–**c** Structuring photoluminescence using different waveguide geometries. **a** Straight waveguide that supports multiple modes in the core, scale bar 20 μm. The red insets are a part of the imaging region on the straight waveguide that is blown up to show the MMI pattern at different instances of time. The orange arrow indicates light propagation direction. Scale bar 5 μm. The schematic diagram shows the geometry and width of the straight waveguide fabricated on a wafer. **b** Four-arm crossing waveguide provides more illumination spatial frequencies, scale bar 20 μm. The green inset is the blown-up region of the imaging area on the four-arm crossing waveguide, showing different speckle patterns at different instances of time, scale bar 5 μm. The green arrows indicate light propagation direction. The schematic diagram shows the geometry and width of the four-arm crossing waveguide. **c** SIM chip where two single-mode waveguides are made to overlap at the imaging area enclosed by the blue inset, scale bar 20 μm. The blown-up regions show the interference fringe pattern at three different phases, 2 μm. The blue arrows indicate light propagation direction. The schematic diagram shows the geometry of the SIM chip for two-dimensional SIM work. **d** By averaging out several MMI patterns, an illumination profile devoid of speckle patterns, as shown in Supplementary Fig. S2b, over a large field-of-view can be generated, scale bar 100 μm. The color grading visible from left to right is attributed to propagation loss (Supplementary Table 1). **e** Ratio of incoherent PL scattering to coherent scattering for a single waveguide at three different wavelengths is plotted. Here, the waveguide is excited at 488 nm, 561 nm and 640 nm, and the corresponding emissions may be detected in FITC, TRITC and CY5 channels. **f** Normalized PL emission with a root mean squared value of 1007 a.u. is plotted here as a function of time. No bleaching was observed for about 2 h of imaging, indicating a stable emission that is suitable for long-term cell imaging. **g** PL emission varies linearly with the input coupling power, implying a linear system

In this article, near-field illumination via the intrinsic incoherent PL of Si_3_N_4_ waveguides is utilized to solve the abovementioned challenges associated with far-field label-free optical microscopy. That is, sample features which are located within 100 nm of the waveguide core scatter the evanescently decaying incoherent PL into the far-field. This incoherent scattering permits achieving label-free super-resolution via fluorescence-based algorithms. Thus, high-contrast label-free super-resolved images can be generated, without photo-toxicity and photobleaching plaguing the imaging process. The developed label-free incoherent imaging system is termed Evanescently decaying photoluminescence scattering enables label-free optical nanoscopy (EPSLON), which builds and extends the concepts outlined by Ruh et al.^28^, Wicker and Heintzmann^13^, and previous work based on photonic-chip microscopy^32^. The concepts of EPSLON, which enable circumventing the Abbe-limit and achieving label-free super-resolution, are explained and experimentally demonstrated in Fig. 1 and Fig. 2. Thus, PL from Si_3_N_4_ waveguide is experimentally validated, as a solution to the challenges associated with far-field label-free optical microscopy, via high-contrast label-free super-resolved images of polystyrene nanobeads, gold nanoparticles, weakly scattering specimens like extracellular vesicles (EVs) and histological samples including rat and kidney tissue sections.

## Results

### Problem statement

Here we describe why fluorescence-based super-resolution algorithms applied to coherently scattering samples do not yield any resolution gain beyond the diffraction limit^13^. In label-free mode, when two particles are illuminated by a monochromatic plane wave, the intensity registered by the camera is  where 〈 〉 represents time averaging by the detector, $h\left(\vec{r}\right)$ is the coherent point spread function of the imaging system, ⨷ represents the convolution operation and the scattered scalar electric fields ${E}_{\mathrm{1,2}}$ are linked to the applied electric fields via polarizability of the two particles^53^. Due to statistical similarity or coherence between the overlapping scattered fields, the intensity registered by the camera is non-linearly related to the particle concentration and is a function of $\Delta \varphi =\vec{k}.\vec{{r}_{2}}-\vec{k}.\vec{{r}_{1}}$. This implies that the image generated by the camera varies with either a change in the illumination angle $\vec{k}$, or with the relative positions of the particles $\vec{{r}_{2}}-\vec{{r}_{1}}$.

Next, to illustrate the image formation process in fluorescence microscopy, the phase objects are replaced with fluorescent molecules. Analogous to the strengths of the scattered fields, ${\left|{a}_{1}\left(\vec{{r}_{1}}\right)\right|}^{2}$ and ${\left|{a}_{2}\left(\vec{{r}_{2}}\right)\right|}^{2}$, are the brightness of the molecules that typically depends on the illumination intensity at the location of the molecule. The molecules can also be assumed to emit independently^54^ and stochastically, typically on the order of nanoseconds^55^. The following properties of these molecules are utilized in fluorescence microscopy by collecting only the Stokes-shifted light emitted by the molecules:

(i) Stochastic emission between the molecules causes the phase difference between the emitted fields to be a function of time, $\varDelta \varphi \left(t\right)$. It implies that the molecule emissions are incoherent with respect to one another, or, in other words, we can say that the transversal coherence length is determined by the size of an individual molecule. This gives rise to similar images for different illumination angles of the incident plane wave. It is this property that allows the usage of structured light in SIM^13,56^ or the intrinsic photo-kinetics of the molecules in IFON algorithms to enhance the resolution^5^,
(ii) Excited lifetime on the order of nanoseconds of these stochastically emitting molecules helps average several random interference patterns within the integration time of the camera, thus helping in mitigating speckle noise.

In addition, the molecular specificity offered by these fluorescent molecules enables multi-color imaging of different cell organelles. It can be concluded that the suppression of speckle noise and molecular specificity offered by the molecules enables high-contrast imaging, and the linear relationship between molecular concentration and image plane intensity helps in enhancing the resolution via fluorescence-based super-resolution algorithms. Therefore, to improve the label-free resolution via fluorescence-based algorithms, we need to ensure that there exists no statistical similarity between the scattered fields originating from different locations^13^. This calls for the mutual intensity to be ${\rm{\delta }}$-function correlated^53^, i.e.,

1 $$J\left(\vec{{r}_{1}},\vec{{r}_{2}}\right)=\left\langle {E}_{T}\left(\vec{{r}_{1}},t\right){{E}^{* }}_{T}\left(\vec{{r}_{2}},t\right)\right\rangle ={K}{I}_{T}\left(\vec{{r}_{1}}\right){\rm{\delta }}\left(\vec{{r}_{1}}-\vec{{r}_{2}}\right)$$

where $J\left(\vec{{r}_{1}},\vec{{r}_{2}}\right)$ is the mutual intensity and determines the spatial correlation of the fields, ${E}_{T}\left(\vec{r},t\right)={E}_{1}\left(\vec{r},t\right)+{E}_{2}\left(\vec{r},t\right)$ is the total field reaching the camera, ${\rm K}$ is a real constant and ${I}_{T}$ is the image generated by the camera. This will ensure an incoherent imaging system. Equation (1) can be assumed to be satisfied in fluorescence microscopy because the transversal spatial coherence length is determined by the size of the fluorescent molecules, and the image generated by the camera indicates the spatial locations of the fluorescent molecules.

Hence, based on the above discussion, to circumvent the label-free diffraction limit using fluorescence-based super-resolution algorithms, we need to develop a light source whose electric field has ${\rm{\delta }}$-function spatial correlations and then acquire an image stack exhibiting intensity fluctuations to apply SIM or intensity fluctuation-based algorithms. This can be realized experimentally via the EPSLON configuration. Figure 1a, b highlights the differences between epi-fluorescence and total internal-reflection-based fluorescence (TIRF) imaging schemes. Figure 1c is label-free epi imaging and is the label-free coherent counterpart of Fig. 1a. Figure 1d is the schematic representation of EPSLON, the proposed label-free incoherent imaging scheme in TIR configuration. The necessity of a TIR imaging scheme arises because it is known that spatial correlation or coherence function increases with propagation distance^57^ and hence, a near-field illumination is necessary to ensure an incoherent system. This restricts the applicability of EPSLON to two-dimensional imaging. Figure 1e is waveguide-based label-free coherent imaging and, Fig. 1f is the waveguide-based label-free incoherent imaging scheme, EPSLON. Next, images acquired in waveguide-based coherent and their corresponding incoherent EPSLON schemes are shown in Fig. 1g, where speckle suppression due to the loss of phase information in the scattered light is evidenced in the EPSLON configuration image. This loss of phase information also implies that identical images are generated for arbitrary illumination angles in EPSLON configuration, as opposed to label-free coherent imaging, where phase differences could result in varying images at the camera plane. This implies that by merely changing the illumination angle in a coherent imaging technique, e.g., Fourier Ptychography, additional information of the sample may be captured at the camera plane. This is in contrast with incoherent imaging techniques like fluorescence microscopy, where no new information may be captured by changing the illumination angle. This concept is demonstrated experimentally in Fig. 1h–j, where the label-free coherent and its corresponding label-free incoherent, EPSLON, images are provided.

### EPSLON: a solution for high-contrast far-field label-free super-resolution microscopy

To employ a light source whose electric field has ${\rm{\delta }}$-function correlations, we resort to the high-index contrast (Δ*n* ≈2) Si_3_N_4_ optical waveguide deposited using plasma-enhanced chemical vapor deposition (PECVD). Waveguide fabrication, properties of the guided modes and their spatial frequency extent are provided in Supplementary Section 2 and in previous works^58,59^. The propagation loss in these waveguides as a function of wavelength is determined and given in Table 1, Supplementary Section 2. The choice of Si_3_N_4_ over other high-index contrast optical waveguides, such as tantalum pentoxide or titanium dioxide, is due to the room-temperature visible PL generated inside the core during the transfer of optical power along its length.

Determining the origin and lifetime of this emission is not within the scope of this work which is a widely researched area^60–62^. It is found to be dependent on the waveguide fabrication scheme employed and could be attributed to the intrinsic fluorescence of the material^14^. The PL emission spectrum is broad (≈500 nm to 900 nm)^15^ and the lifetime of these states is found to vary on the order of a few nanoseconds to a few hundred microseconds, depending on the origin of the PL^16,63,64^. Such an emission could be visualized as a very large number of fluorescent molecules embedded in a material and emitting stochastically. Hence, if this PL is used for near-field illumination of samples, then Eq. (1) will be satisfied for the incoherently scattered fields.

The next problem to tackle is generating an image stack with intensity fluctuations for the application of fluorescence-based algorithms. Structuring the illumination beam and manipulating the photophysical properties of the fluorescent molecules are some of the methods typically employed to generate image stacks with intensity fluctuations. In EPSLON, this problem is resolved by resorting to Si_3_N_4_ waveguides of the following types: (1) Straight waveguides with strip geometry and large widths that support a large number of the guided modes, generating MMI patterns (Fig. 2a)^32^, (2) four-arm junction multi-mode strip waveguides for speckle illumination from different azimuthal angles (Fig. 2b)^32^ and, (3) a single mode SIM chip with rib geometry and phase modulation for one-dimensional structured illumination (Fig. 2c)^65^, and four-arm junction multi-mode strip waveguide for two-dimensional structured illumination. Simulation analysis and experimental results are presented in the Supplementary Section 3 to validate how multi-mode illumination patterns, when used in tandem with IFON, help gain resolution. Our results in Supplementary Figs. S3–S6, show that the different azimuthal illumination frequencies in a four-arm junction multi-mode waveguide help achieve low correlation between different scatterers and thereby aid IFON techniques like SOFI^5^ and SACD^9^ that exploit intensity correlations between different emitters to generate super-resolved images.

### Image formation process in EPSLON

A laser is coupled into a Si_3_N_4_ waveguide via a microscope objective MO_1_ (Supplementary Fig. S7). The coupled optical power is distributed among multiple coherent guided modes, which correspond to the eigen vectors of the guiding structure. As the modes guide power along the length of the waveguide, they also induce a broadband PL along the length as shown in Fig. 2d, e. The Si_3_N_4_ waveguide employed in this work demonstrated PL in all the commonly used wavelengths in bio-imaging: 488 nm, 561 nm, and 647/660 nm (Fig. 2e). This PL of Si_3_N_4_ waveguide does not exhibit any bleaching effect over long periods of time (Fig. 2f), unlike the autofluorescence in polymer waveguides^66^, and it varies linearly with the excitation powers used in the experiments here (Fig. 2g).

Now, to explain the origin of fluctuations in intensity and how a uniform illumination can be generated, consider the case of a multi-mode straight waveguide. Fluctuations over time, ${I}_{\rm{core}}^{m}\left(\vec{r,}t\right)$, can be induced by oscillating MO_1_ using a piezo-stage across the input facet of the waveguide, which excites different sets of modes ${\psi }_{m}\left(\vec{r},t\right)$ with relative amplitudes $0\le {a}_{m}(t)\le 1$, see Supplementary Fig. S7. The instantaneous PL intensity at each location in the core depends on the coherent superposition of the modes and can be represented mathematically as ${I}_{{core}}^{m}\left(\vec{r,}t\right)=\eta (\lambda ){\left|{\sum }_{m}{a}_{m}(t){\psi }_{m}\left(\vec{r},t\right)\right|}^{2}$, where *η*(*λ*) is assumed to be a constant across the material for a specific wavelength.  corresponds to the scalar representation of the *m*^th^ guided mode with fixed transversal profile *E*_*m*_ (*x, y*) and propagation constant *ß*_*m*_. As the PL emission occurs inside a high-index core, a part of the PL light is confined to the core due to total internal reflection at the core–cladding interface, while the remaining part gets transmitted into the far field, which is visible as an omnipresent background or noise. This ratio is quantified experimentally and found to be about 0.01, Supplementary Fig. S8a. This implies that the scattered light off the sample in EPSLON configuration can be stronger than the background. This may also be understood from Supplementary Fig. S8b, where tissue scattering is predominantly due to confined light in the waveguide.

This evanescently decaying light at the core-cladding interface can propagate into the far-field when there exists an index perturbation, for example, in the presence of a sample at the core–cladding interface (Fig. 1d). Hence, this technique is abbreviated as EPSLON. That is, both the coherent and the Stokes-shifted incoherent PL light are scattered into the far field. By invoking a first-order Born approximation for evanescent field excitation of biological specimens $S\left(\vec{r}\right)={\iint }_{S}\alpha ({\vec{r}}_{k})\delta \left(\vec{r}-{\vec{r}}_{k}\right)d{\vec{r}}_{k}$ with α being the polarizability, it is seen that these scattered fields contain the information of the sample. Only these scattered fields are collected by the microscope objective MO_2_ and relayed onto the camera via a tube lens because of the decoupled illumination and detection scheme offered by waveguides (Supplementary Fig. S7). Through the usage of appropriate bandpass filters, the coherently scattered light gets filtered out, and only the incoherent light is detected. The oscillation of MO_1_ is synchronized with the detector so that an image is acquired at each excitation point of the waveguide. By invoking Eq. (1) and neglecting noise, an EPSLON image (Fig. 1d) at the camera plane can generally be described mathematically as2

The above concepts and experimental results can be summarized as follows: (i) Speckle noise is mitigated in EPSLON images as opposed to label-free waveguide-based coherent images due to stochastic fluctuations between the scattered fields, Fig. 1g. (ii) Stochastic fluctuations imply that phase relationships between the scattered fields are lost in EPSLON images as opposed to label-free coherent images. This also leads to identical images for different illumination angles, Fig. 1h–j. (iii) Intensity–fluctuations are induced over time due to the time dependence of ${a}_{m}(t)$ and ${\psi }_{m}\left(\vec{r},t\right)$. This is evident in the line plots in Fig. 1j and in the MMI patterns shown as insets in Fig. 2a–c. This is also validated using simulations in Supplementary Section 5, Fig. S9. Thus, EPSLON, when used in tandem with fluorescence-based super-resolution algorithms, helps develop a high-contrast incoherent label-free super-resolution imaging system.

### Experimental results: applications of EPSLON

**Fig. 3:**
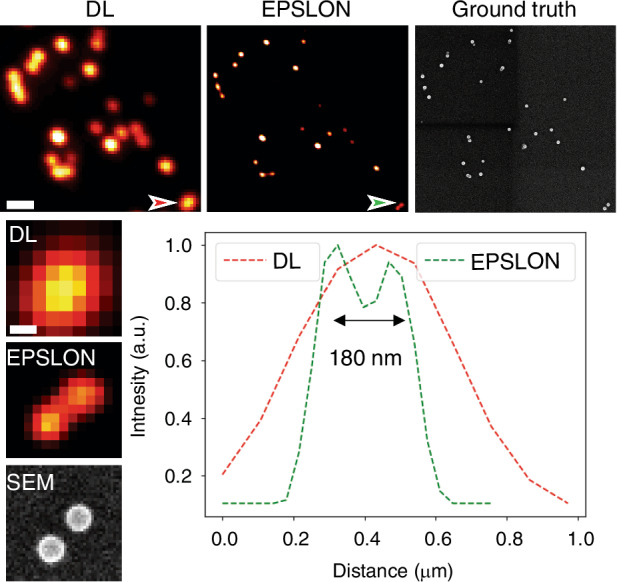
EPSLON via SACD for label-free super-resolution imaging of 100 nm polystyrene beads using straight waveguides. Diffraction-limited DL image, super-resolved EPSLON image and scanning electron microscope (SEM) ground truth image are shown, scale bar 1 µm. The red and green line plot corresponds to intensity variations along the red and green arrows in the DL and EPSLON images, respectively. The blown-up images show unresolved beads in the DL image, resolved beads in the EPSLON image and the validation via the ground truth SEM image, scale bar 100 nm

The potential of EPSLON is initially demonstrated on nanobeads. A straight waveguide is employed, and the images of 100 nm polystyrene beads placed directly on top of the waveguide core are acquired using a detection MO with numerical aperture NA = 0.9, Fig. 3a. For brevity, the diffraction-limited label-free image and its corresponding reconstructed super-resolved image in the following sections are termed DL and EPSLON, respectively. The acquired image stack of 100 frames is then given as input to a fluorescence-based super-resolution algorithm called as super-resolution method based on auto-correlation two-step deconvolution (SACD)^9^. The choice of SACD over other IFON algorithms is based on the simulation studies shown in Supplementary Section 3, and due to the better performance of the algorithm at low signal-to-background ratios^67^. Now to validate the super-resolved images generated by SACD, the same sample is imaged by a scanning electron microscope (SEM), which serves as the ground truth image. In Fig. 3a, the line profiles indicate the intensity variations across the particles in the insets. The line profile of the green inset clearly indicates that EPSLON resolves the unresolved nanobeads shown in the red inset in the DL image. The peak-to-peak distance between the beads is 180 nm. It is seen that EPSLON and the SEM images, i.e., the super-resolved and the ground truth images, agree. Then, Supplementary Fig. S10 to Fig. S12, the potential of EPSLON using various waveguide geometries is demonstrated: four-arm junction waveguide and single-mode SIM chip for 2D and 1D SIM, respectively. In Supplementary Fig. S10, 195 nm polystyrene beads are placed on top of a four-arm junction waveguide, and images are acquired using a 0.75 NA detection objective. This geometry helps achieve 2D label-free SIM. Additionally, a smaller region within this same field-of-view is also imaged using a 0.9 NA detection objective, and the DL and EPSLON images are shown in Supplementary Fig. S11. Finally, in supplementary Fig. S12, we demonstrate label-free 1-D SIM using a SIM chip^65^. Also, the dependence of speckle size and resolution on ${\lambda }_{{ex}}$, $\theta$, and ${\lambda }_{\det }$ is experimentally demonstrated and verified with the theoretical predictions. It is experimentally shown that the period of the generated fringes depends on ${\lambda }_{{ex}}$ and $\theta$, and the resolution of the DL image so generated depends in addition also on ${\lambda }_{\det }$. This is illustrated in Supplementary Section 8 (Supplementary Fig. S13–Fig. S16).

**Fig. 4:**
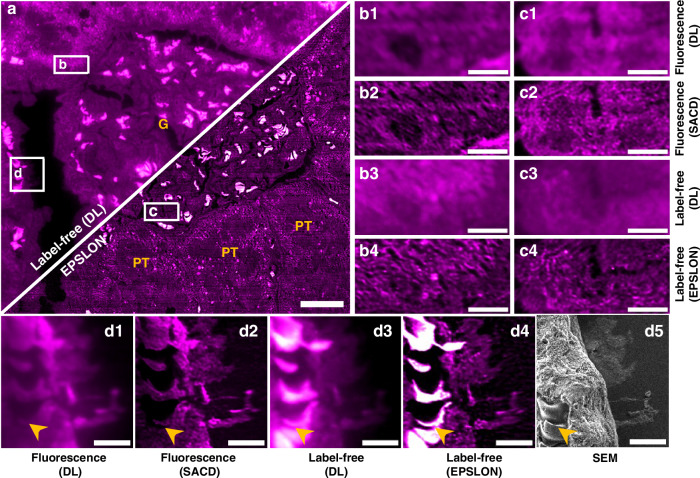
EPSLON via SACD for label-free super-resolution imaging of rat kidney tissue sections and benchmarking via correlative microscopy, EPSLON-TIRF and EPLSON-SEM. **a** Label-free diffraction-limited (DL) and its corresponding super-resolved EPSLON images are shown. The region ‘G’ labeled in yellow font indicates Glomerulus, and the regions labeled ‘PT’ in yellow font indicate Proximal Tubuli of the kidney section. Three regions of interest enclosed in white dotted boxes are labeled ‘b’–‘d’. EPSLON image is gamma corrected and displayed. Scale bar 25 µm. **b1**, **c1**, **d1** Diffraction-limited TIRF images of the regions enclosed by white dotted boxes in **a** are blown up and shown, scale bar 5 µm. **b2**, **c2**, **d2** Corresponding super-resolved TIRF images of regions in (**b1**, **c1**, **d1**) are shown, scale bar 5 µm. **b3**, **c3**, **d3** Label-free DL images of regions enclosed by the white dotted boxes in **a** are shown magnified, scale bar 5 µm. **b4**, **c4**, **d4** Super-resolved EPSLON images of regions corresponding to (**b3**, **c3**, **d3**) are shown, scale bar 5 µm. **d5** SEM image of the region enclosed by the white dotted box labeled ‘d’ is shown. The yellow arrowheads denote the location of red blood cells, being imaged throughout all the microscopy methods. Scale bar 5 µm. The mean FRC resolution of the label-free DL image is 256 nm, and the mean FRC resolution of EPSLON image is 133 nm

Next, to showcase the potential of EPSLON for life sciences, we chose biological samples such as EVs (Supplementary Section 9), and histological samples from diverse species, including rat kidney, as well as human kidney and placental tissue sections. Firstly, the utility of EPSLON for super-resolution imaging of kidney tissue is discussed. The ultrastructural analysis of this organ is crucial for the identification of several renal pathologies, such as minimal change disease^68^. While significant attention has been given to fluorescence-based super-resolution optical methods for kidney research and diagnosis in recent years^69^, the utility of label-free optical super-resolution approaches remains largely unexplored, making EPSLON a promising method for this field. In this work, rat kidney sections are used as test samples, as shown in Fig. 4. 250 frames of rat kidney sections are imaged first in label-free mode, then in waveguide TIRF mode (i.e., using fluorescence markers), and finally using SEM. Figure 4a shows the label-free DL and EPSLON images of a rat kidney section acquired using a detection MO with NA = 1.42. The central portion of the image labeled ‘G’ in yellow font represents a kidney glomerulus, while regions labeled ‘PT’ in yellow font correspond to the proximal tubuli in the kidney section. Three regions of interest labeled ‘b’–‘d’ in Fig. 4a are blown up and shown. Figure 4b1–c1 are TIRF-DL images of regions enclosed in ‘b’ and ‘c’. Their corresponding TIRF-SACD images are shown in Fig. 4b2–c2, respectively. Label-free DL images of regions enclosed in ‘b’ and ‘c’ are shown in Fig. 4b3–c3. Their corresponding EPSLON images are shown in Fig. 4b4–c4, respectively. Fig. 4d1–d2 are TIRF-DL and TIRF-SACD images of the region labeled ‘d’. Label-free DL and EPSLON images of the region labeled ‘d’ are shown in Fig. 4d3–d4, respectively. A SEM image of the same region is shown in Fig. 4d5. It must be noted that EPSLON is an evanescent wave illumination technique; therefore, the EPSLON images show structures that are within the penetration depth of the evanescent field, i.e., structures in proximity to the photonic-chip surface are imaged. SEM image, on the contrary, showcases structures on the top of the sample, visualizing the part of the sample opposite to the photonic-chip surface. Supplementary Movie 1 compares DL and EPSLON at different regions of interest in Fig. 4. The resolution of optical images acquired is quantified using FRC^70^. The mean resolution of the label-free image is 256 nm for DL and 133 nm for EPSLON, which implies a $\sim$1.92-fold resolution gain. The local FRC plot of the whole field-of-view presented in Fig. 4 is shown in Supplementary Fig. S19.

**Fig. 5:**
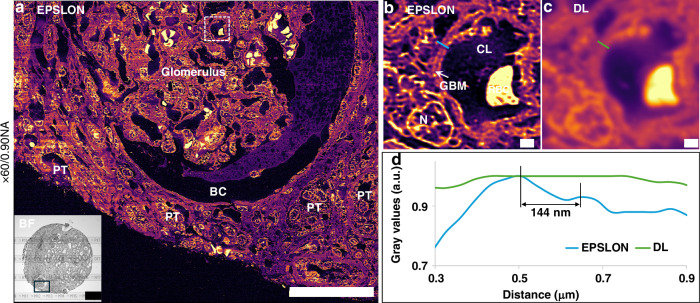
EPSLON via SACD for human kidney histopathology. **a** Label-free super-resolved EPSLON image of a human kidney tissue section is shown, scale bar 50 µm. A mean FRC resolution of 129 nm is observed. The gray scale inset is the bright field image of the kidney section imaged with a 4×/0.10 NA detection objective, scale bar 200 µm. The pseudo-color image illustrates the EPSLON results of the selected glomerular region in the BF image enclosed within the black box. EPSLON provides a high-contrast contextual visualization of the sample, enabling the identification of structures such as the glomerulus, the Bowman’s capsule (BC), and many adjacent proximal tubuli (PT). **b** The region in the EPSLON image enclosed within the white dotted box is blown up and shown, scale bar 1 µm. This figure reveals microanatomical structures including a glomerular basement membrane (GBM), a capillary lumen (CL) with a red blood cell (RBC), and the nucleus (N) of an adjacent podocyte. **c** The corresponding diffraction-limited (DL) view of the FOV in (**b**) is shown, scale bar 1 µm. Note the poor contrast and resolution as compared to the EPSLON image. **d** Line profile measurements over the GBM reveal a lateral resolution of approximately 144 nm using EPSLON, which cannot be measured in DL modality. The blue line represents intensity variation in the EPSLON image along the region shown in (**b**), and the green line represents intensity variation along the DL region shown in (**c**)

Another potential application of EPSLON in medical sciences is to facilitate the screening of diseases that require high-resolution visualization over large areas for accurate diagnosis. An example of this is kidney histopathology, where light microscopy is first used for contextual examination of the tissue microanatomy, followed by electron microscopy to visualize ultrastructural details not visualizable via conventional optical microscopy. The applicability of EPSLON in clinical settings is demonstrated by analyzing a human kidney sample preserved by the standard formalin-fixation and paraffin-embedding methodology^71^. After identifying a glomerular area using bright field modality at low magnification, see inset in Fig. 5a, the region of interest was further inspected in EPSLON at 60× magnification using an M.O. with a NA = 0.9. The resulting image, shown in panel Fig. 5a, offers a large field-of-view (FOV) high-contrast view of the intricate anatomy of the glomerulus and its surrounding tissue including the Bowman’s capsule (BC), and many adjacent proximal tubuli (PT). A zoom-in visualization over a glomerular capillary, presented in Fig. 5b, allows for a clear observation of relevant structures such as the glomerular basement membrane (GBM) between a capillary lumen (CL) containing a red blood cell (RBC), and a surrounding podocyte cell with its distinctive nucleus (N). The mean FRC resolution of the EPSLON image is 129 nm. The GBM thickness is a key indicator for the diagnosis of several renal conditions, such as thin basement membrane disease and Alport syndrome^72^, involving GBM thinning, or primary membranous nephropathy^73^, with GBM thickening. In clinical settings, this microanatomical feature is usually measured using ultrathin polymer-embedded tissue sections in transmission electron microscopy. In this example, a line profile measurement over the GBM reveals a lateral resolution down to 144 nm in EPSLON, blue line in Fig. 5b, d, allowing for an optical sub-diffraction limit measurement on a conventional paraffin-embedded sample. The GBM region is not discernible in the DL image counterpart, green line in Fig. 5c, d. Also, another region of interest in the same FOV is presented in Supplementary Fig. S20. Finally, in Supplementary Fig. S21 and Supplementary Fig. S22, we showcase the potential of EPSLON for the evaluation of human placental tissue sections using straight waveguides in tandem with SACD.

## Discussion

Fluorescence-based algorithms have been previously applied to coherently scattering specimens^74^. However, the reconstructed images generated must be interpreted with caution with regard to resolution gain beyond the diffraction limit, as coherence of the scattered light cannot be neglected^13,32^. A recent review outlines the working principles and challenges associated with these and various other label-free optical microscopy techniques^75^. In this proof-of-concept work, EPSLON, this issue of coherence is circumvented for two-dimensional samples by realizing a light source whose electric field has ${\rm{\delta }}$-function correlations, i.e., by using the PL property of Si_3_N_4_ waveguides for near-field illumination of unlabeled samples. Additionally, PL emission takes place within the core matrix, and a part of it gets transmitted into the far field. This prevents realizing an ideal chip-based imaging system where only the scattered light off the sample reaches the camera, and therefore may have practical limitations. That is, even though the IFON algorithms in principle can achieve unlimited spatial resolution^5,9^, a poor signal-to-background ratio, correlations between various scattering points arising due to the MMI patterns and photo-physics of the PL emission process can affect the performance of the reconstruction algorithm to generate super-resolved images with fidelity. In this work, despite the limited photon budget, we have demonstrated proof-of-concept label-free super-resolution results on nanobeads, EVs, human placenta tissue, human kidney tissue and rat kidney sections. The experimental particulars are provided in Table 2 and Table 3 in the Supplementary Material. Also, Supplementary Fig. S23 showcases resolution gain in EPSLON over some of the commonly employed deconvolution algorithms^76^. An immediate focus for the near future is to understand and engineer the blinking rate of the PL process in Si_3_N_4_ to improve the achievable spatial resolution, so that the intrinsic blinking in the emission process itself is utilized. This will enable illumination of the unlabeled sample with a uniform intensity profile as demonstrated in ref.^32^, instead of relying on the MMI patterns to induce fluctuations as demonstrated here and in ref.^17^. In future works, we aim for novel chip designs^77,78^ which will improve the signal-to-background ratio. The other challenge of a lack of specificity in label-free imaging can be mitigated by resorting to machine learning based tools^79^.

This work lays the foundation for synthesizing a label-free incoherent imaging system compatible with the myriad of fluorescence-based super-resolution algorithms to circumvent the spatial diffraction limit. It is expected that EPSLON will trigger further developments of label-free super-resolution incoherent optical microscopy methods and their application in biology, with particular attention to histological analyses where a fast, simple, cost-effective, and high-resolution imaging method is beneficial for medical guidance and diagnosis. Future studies will also investigate the role of intrinsic autofluorescence of tissue sections in label-free imaging. Expanding the concepts of EPSLON to be applied in tandem with other fluorescence-based super-resolution algorithms like non-linear SIM^54^ and STED^80^ is currently in progress. This work could also initiate further developments within integrated optics to harness the PL properties of different materials. Interestingly, PL in waveguides is an undesirable phenomenon as it increases the propagation losses of the guiding structures. However, in this work, this intrinsic incoherent PL of Si_3_N_4_ waveguide is harnessed for near-field illumination to develop an incoherent imaging system that surpasses the Abbe limit and generates super-resolved label-free images via fluorescence-based super-resolution algorithms.

## Materials and methods

A 2 µm-thick oxide layer was thermally grown on a silicon wafer, followed by the deposition of a 150 nm thick Si3N4 layer using plasma-enhanced chemical vapor deposition (PECVD). The 2D channel waveguides were defined by photolithography and etched using reactive ion etching (RIE). Then, a silicon oxide layer was deposited using PECVD on the patterned nitride layer for protection. Finally, the oxide layer was patterned and removed from certain regions of each waveguide to create the imaging regions. The oxide layer was removed using a combination of both dry and wet etching^58^.

A laser (iChrome MLE, Toptica Photonics) is coupled and guided inside a single-mode fiber (P1-460Y-FC-1, Thorlabs) held on a single-axis X-piezo stage by a vacuum chuck is collimated via a collimator (RC04FC-P01, Thorlabs) and directed to the back-focal plane of a coupling objective (MO1—Olympus LMPanFL N 50×/0.5 NA). The light is focused by MO_1_ and coupled into the Si_3_N_4_ waveguide mounted on a three-axis XYZ nanometer precision stage (Nanomax 300, Thorlabs). The coupled laser light in the waveguide induces a broadband photoluminescence PL in its core. Any index perturbation scatters both the guided coherent, shown in blue, and the incoherent PL light, shown in red, into the far-field via detection objective MO2, passing a tube lens with spectral filters (BLP02-561R-25, Stock#67-034; BLP01-664R-25, FF01-692/40-25, SEMROCK) onto a sCMOS Hamamatsu C13440-20CU camera. The spectral filters are chosen to reject the coherent laser light and transmit only the incoherent PL light. The schematic of the experimental setup is shown in Supplementary Fig. 7.

## Supplementary information

Supplementary

Demonstrates correlative EPSLON and SEM imaging of human placenta tissue sections. The movie is associated with the Supplementary Fig. 21

Demonstrates correlative EPSLON and diffraction limited imaging of rat kidney tissue section. The movie is associated with the Fig. 4

## Data Availability

The data used in this study are available from the corresponding authors upon reasonable request.
