# Supplementary material for: Chip-based label-free incoherent super-resolution optical microscopy
Source: Light Sci Appl. 2025 Aug 4;14:259. doi: 10.1038/s41377-025-01914-x (PMC12319112; doi:10.1038/s41377-025-01914-x)
Supplement: Supplementary file 1 — Supplementary [file 41377_2025_1914_MOESM1_ESM.docx]

**Supplementary Information for**

**Chip-based label-free incoherent super-resolution optical microscopy**

Nikhil Jayakumar**^1^, Luis E. Villegas-Hernández^1^, Weisong Zhao^2^, Hong Mao^1^, Firehun T Dullo^3^, Jean-Claude Tinguley^1^, Krizia Sagini^4,5^, Alicia Llorente^,4,5,6^, Balpreet Singh Ahluwalia*^1,7^

^1^*Department of Physics and Technology, UiT The Arctic University of Norway, Tromsø, 9037, Norway*

^2^*Innovation Photonics and Imaging Center, School of Instrumentation Science and Engineering, Harbin Institute of Technology, Harbin, China*

^3^*Department of Microsystems and Nanotechnology, SINTEF Digital, Gaustadalleen 23C, 0373 Oslo, Norway*

^4^ *Department of Molecular Cell Biology, Institute for Cancer Research, Oslo University Hospital, The Norwegian Radium Hospital, 0379 Oslo, Norway*

*^5^Centre for Cancer Cell Reprogramming, Faculty of Medicine, University of Oslo, Montebello, 0379 Oslo, Norway*

*^6^Department for Mechanical, Electronics and Chemical Engineering, Oslo Metropolitan University, Oslo, Norway*

*^7^Department of Clinical Science, Intervention and Technology, Karolinska Institute, Sweden*

[*Balpreet.singh.ahluwalia@uit.no](mailto:*Balpreet.singh.ahluwalia@uit.no), [**nik.jay.hil@gmail.com](mailto:**nik.jay.hil@gmail.com)

**1. Label-free high-resolution microscopic techniques**

Below we have categorized the state-of-the-art high-resolution label-free techniques into three categories:

Technique 1: Techniques that use the concept of synthetic aperture/spatial frequency shift for coherently scattering samples

Technique 2: Synthetic aperture/Spatial-frequency shift concepts for coherently scattering samples using chip-based solutions

Technique 3: Techniques that apply fluorescence-based algorithms to coherently scattering samples.

Technique 1: High-resolution techniques that use the concept of synthetic aperture for label-free microscopy

Abbe’s resolution-limit when considering oblique illumination for elastically scattered light is $\frac{\lambda_{ill}}{{NA}_{ill}+{NA}_{det}}$. And in fluorescent microscopy, because of the emission properties of fluorescent molecules, the Abbe resolution limit is $\frac{\lambda_{det}}{2{NA}_{det}}$. Below we highlight the state-of-the-art label-free optical techniques and briefly elucidate their working principles. This will help to understand the differences between EPSLON and label-free state-of-the-art optical techniques.

1. Cotte, Yann, et al. "Marker-free phase nanoscopy." *Nature Photonics* 7.2 (2013): 113-117.
2. Zheng, Guoan, Roarke Horstmeyer, and Changhuei Yang. "Wide-field, high-resolution Fourier ptychographic microscopy." *Nature photonics* 7.9 (2013): 739-745.
3. Maire, Guillaume, et al. "Phase imaging and synthetic aperture super-resolution via total internal reflection microscopy." *Optics letters* 43.9 (2018): 2173-2176.
4. Jünger, Felix, Philipp V. Olshausen, and Alexander Rohrbach. "Fast, label-free super-resolution live-cell imaging using rotating coherent scattering (ROCS) microscopy." *Scientific reports* 6.1 (2016): 30393.
5. Jayakumar, Nikhil, et al. "Multi-moded high-index contrast optical waveguide for super-contrast high-resolution label-free microscopy." *Nanophotonics* 11.15 (2022): 3421-3436.
6. Yurdakul, Celalettin, et al. "High-throughput, high-resolution interferometric light microscopy of biological nanoparticles." *ACS nano* 14.2 (2020): 2002-2013.

Ref. [a]: different holograms corresponding to different illumination directions on the sample plane are recorded. After post-processing, a high-resolution image is obtained which is diffraction-limited in Abbe’s sense. The best theoretical resolution after post-processing will be 𝜆_ill_/(NA_illu_ + NA_det_), where 𝜆 is the wavelength of the detected light, NA_illu_ is the numerical aperture of the illumination objective and NA_det_ is the numerical aperture of the detection objective.

Ref. [b]: typically, in Fourier Ptychography, an LED array is used to provide oblique illumination at the sample plane. Then using a phase retrieval algorithm from the intensity images, a high-resolution final image is generated. The final image is still limited by Abbe’s diffraction limit.

Ref. [c]: several azimuthally varying illuminations at the sample plane is provided to perform synthetic aperture reconstruction of the sample. The final resolution is determined by Abbe’s diffraction limit.

Ref. [d]: a 2π azimuthal scan of a laser beam at the back-focal plane of the illumination objective within the integration time of the camera generates a high-contrast image. The final resolution is limited by Abbe’s diffraction limit.

Ref. [e]: a high index contrast optical waveguide is used as the condenser. Multiple modes are excited and averaged within the integration time of the camera. Also, using a four-arm waveguide introduces additional illumination frequencies, but still the imaging system is only partially coherent as not all the illumination frequencies are present and, the phase relationship between the different scatterers is not completely random due to finite coherence area defined by the Abbe limit. This implies that resolution beyond the Abbe limit cannot be achieved using this scheme via fluorescence-based super-resolution algorithms.

Ref. [f]: sample is illuminated via a series of oblique illuminations. The scattered light off the sample and specular reflection from the SiO_2_ substrate helps create a common-path interferometry configuration. Via post-processing a two times resolution over head-on illumination 𝜆_ill_/NA_det_ is obtained. The final resolution is within Abbe’s diffraction limit.

Techniques 2: High-resolution label-free chip-based techniques with concepts like synthetic aperture microscopy

The concepts elaborated in Technique 1 are translated to chip-based platforms here. Hence, an even higher resolution in principle is possible due to the higher refractive-index of the material employed. The resolution achievable is given by Abbe, $\frac{\lambda_{ill}}{{NA}_{ill}+{NA}_{det}}$. All these techniques mitigate coherent speckle noise, either by summing up different speckle patterns on intensity-basis at the camera plane or by using a broadband light source

1. Ströhl, Florian, et al. "Super-condenser enables label free nanoscopy." *Optics express* 27.18 (2019): 25280-25292.
2. Liu, Xiaowei, et al. "Fluorescent nanowire ring illumination for wide-field far-field subdiffraction imaging." *Physical Review Letters* 118.7 (2017): 076101.
3. Tang, Mingwei, et al. "High‐Refractive‐Index Chip with Periodically Fine‐Tuning Gratings for Tunable Virtual‐Wavevector Spatial Frequency Shift Universal Super‐Resolution Imaging." *Advanced Science* 9.9 (2022): 2103835.
4. Pang, Chenlei, et al. "On‐Chip Super‐Resolution Imaging with Fluorescent Polymer Films." *Advanced Functional Materials* 29.27 (2019): 1900126.

Ref. [a]: samples are illuminated in multiple azimuthal directions via evanescent waves generated by a Si_3_N_4_ waveguide. A post-processing algorithm is then applied to generate a high-resolution label-free image. This resolution is determined by Abbe’s diffraction limit of 𝜆_ill_/(NA_illu_ + NA_det_).

Ref. [b]: In this work, broadband light emitted by the nanowire ring (NWR) is coupled into the film waveguide via a single mode. The shortest Stokes shifted wavelength emitted by the NWR will then determine the smallest coherence length. What it essentially implies is that the scattered light will have a constant phase relationship between different locations excited by this mode. Or in other words, the phase information is still preserved in the scattered light. Hence, in principle this technique is not suitable to be applied to in tandem with fluorescence based super-resolution algorithms like structured-illumination microscopy. This is contrary to EPSLON where incoherent imaging system is proposed.

Ref. [c]: a photonic-chip made of Gallium Phosphide (refractive index > 3) is used in this work. Evanescent waves are used for illuminating the sample. Using a reconstruction algorithm a very high-resolution image limited by Abbe’s diffraction-limit can be generated. However, the technique is still coherent in nature and therefore, fluorescence based super-resolution algorithms cannot be applied to circumvent the far-field diffraction-limit. This paper demonstrates 𝝀/4.7 resolution in the label-free mode. Here, authors used objective lens of N.A._det_= 1.49 and GaP chip is used for illumination, i.e., refractive index 3.3 = N.A._illum_. Then Abbe’s resolution limit is 𝜆_ill_/(NA_illu_ + NA_det_) = 𝝀_ill_/4.79.

Ref. [d]: This is also a coherent imaging technique where the sample is illuminated from multiple directions using evanescent waves generated by a waveguide. The broadband light emitted by F8BT is efficiently coupled into waveguides. The guided light then provides evanescent wave illumination for the sample. By illuminating the sample from multiple orientations, a high-resolution image is generated. The resolution of the final image is still limited by Abbe’s diffraction limit. This paper demonstrates 𝝀/3 resolution in the label-free mode. Here, authors used objective lens of N.A._det_= 0.85 and TiO_2_ chip is used for illumination, i.e. refractive index 2.2 = N.A._illum_. Abbe’s resolution limit is therefore 𝜆/(NA_illu_ + NA_det_) = 𝝀/3.05.

Techniques 3: Techniques that apply fluorescence-based algorithms to coherently scattering samples.

1. Lee, Yeon Ui, et al. “Hyperbolic material enhanced scattering nanoscopy for label-free super-resolution imaging.” *Nature communications* 13.1 (2022): 6631.

Here, fluorescence-based super-resolution algorithm BlindSIM is applied in label-free mode. In this work, organic hyperbolic materials are employed to create sub-diffraction sized speckles. The illuminating field is passed through a vibrating multi-mode fiber to create temporal variations. Multiple oblique illuminations in the azimuthal plane are applied at the sample plane. Then Blind-SIM is applied to generate a super-resolved image. The technique resolution is given by Abbe’s limit for elastically scattered light, 𝜆_ill_/(NA_ill_ + NA_det_). Here NA_ill_ is exceptionally high due to the high-index organic hyperbolic material used which leads to a super-resolution. A true incoherent system is imperative to avoid the caveats linked to the application of fluorescence based super-resolution algorithms for the coherent imaging [Wicker, Kai, and Rainer Heintzmann. *Nature Photonics* 8.5 (2014): 342-344, Jayakumar et al. *Nanophotonics* 11.15 (2022): 3421-3436].

1. Dong, Biqin, et al. "Superresolution intrinsic fluorescence imaging of chromatin utilizing native, unmodified nucleic acids for contrast." *Proceedings of the National Academy of Sciences* 113.35 (2016): 9716-9721.

In this work, the intrinsic autofluorescence of the cells mimic the stochastic fluctuations from a fluorescent molecule and therefore, a fluorescence-based localization technique is applied to circumvent the diffraction-limit. This work depends on samples that have unique intrinsic autofluorescence and it is thus not universal to diversified specimens.

The novelty in EPSLON is in using incoherent point-like light sources for near-field illumination of unlabeled samples, i.e., photoluminescence of Si_3_N_4_. It implies that each location of the sample, within the penetration depth of the incoherent evanescent field, scatters the non-propagating incoherent field into the far-field. This permits the application of fluorescence-based algorithms like SIM and IFON in the label-free regime to circumvent Abbe’s diffraction-limit. Such an illumination technique is imperative to avoid the caveats linked with coherent imaging [Wicker, Kai, and Rainer Heintzmann. *Nature Photonics* 8.5 (2014): 342-344, Jayakumar et al. *Nanophotonics* 11.15 (2022): 3421-3436].

**2. Waveguide fabrication**

A 2 µm thick oxide layer was thermally grown on a silicon wafer, followed by the deposition of 150 nm thick Si3N4 layer using plasma enhanced chemical vapor deposition (PECVD). The 2D channel waveguides were defined by photolithography and etched using reactive ion etching (RIE). Then, a silicon oxide layer was deposited using PECVD on the patterned nitride layer for protection. Finally, the oxide layer was patterned and removed from certain regions of each waveguide to create the imaging regions. The oxide layer was removed using a combination of both dry and wet etching [1]. Thus, at the imaging region the specimen is in direct contact with the waveguide core layer, accessing the evanescent field.

**Waveguide modes**


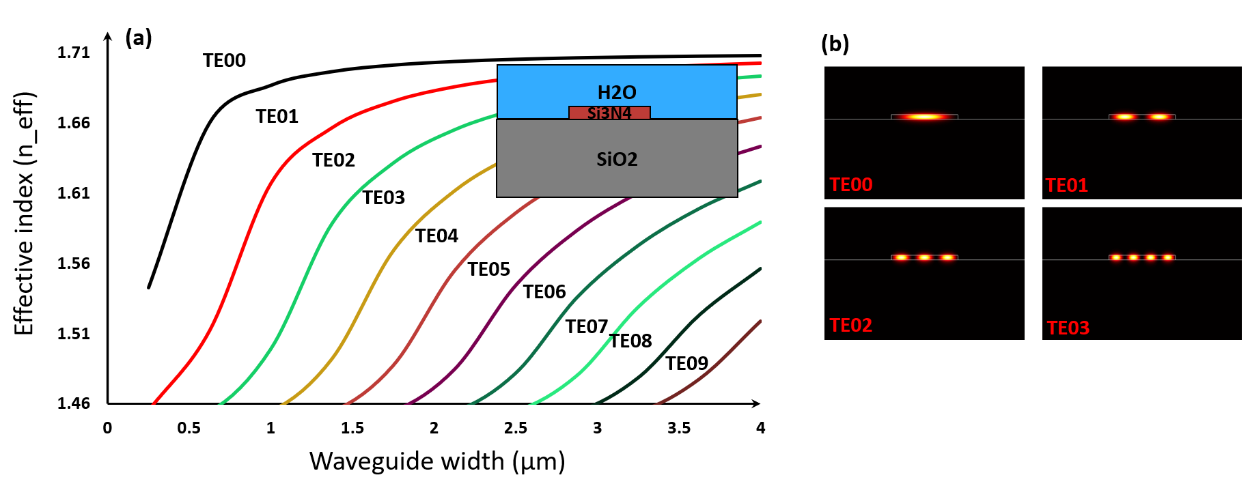


**Figure. S1**: (a) Effective indices of the guided modes for various Si_3_N_4_ waveguide widths. A schematic diagram of the cross-section of the waveguide is provided as an inset in the plot. (b) Mode profiles for the fundamental and higher-order TE - modes. The waveguide in the simulation model is 150 nm thick and 0.25 - 4 µm wide. The guided modes for a Si_3_N_4_ strip waveguide were simulated using the commercial software FIMMWAVE (Photon Design, Oxford, UK), and its effective indices were calculated using the full-vectorial film mode matching (FMM) method.


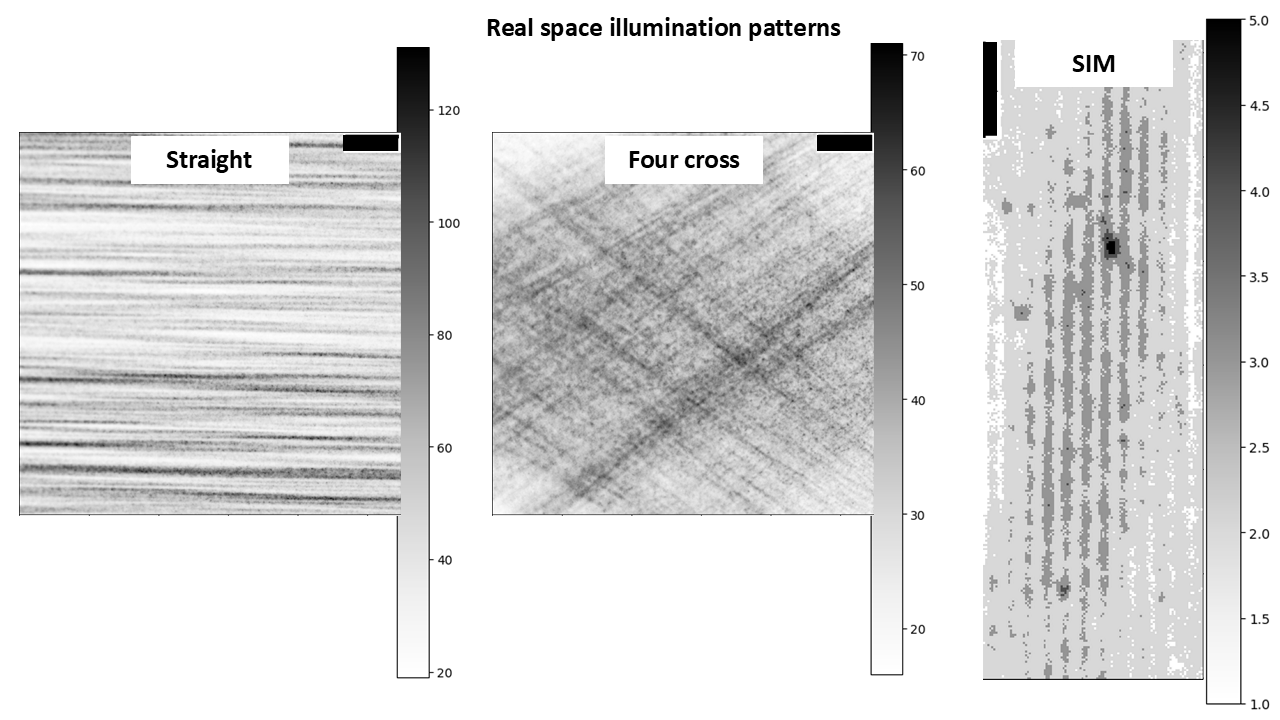


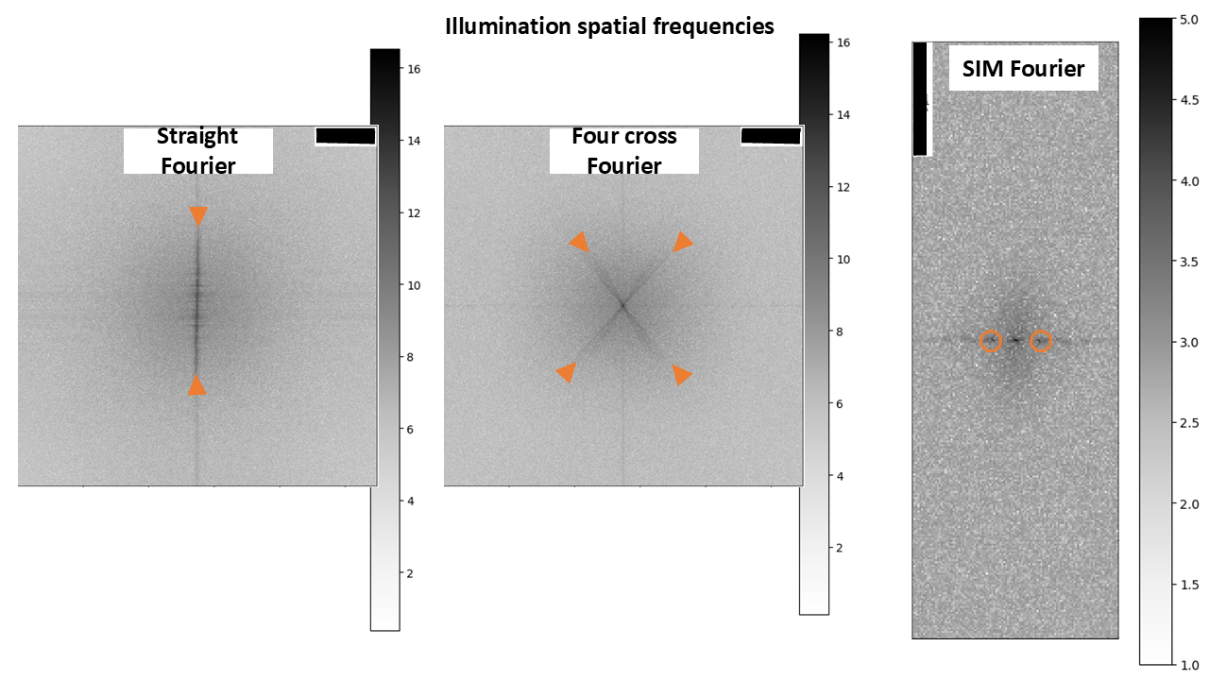


**Figure. S2a: MMI patterns in waveguide geometries employed in EPSLON for beam shaping and their corresponding illumination frequencies.** The multi-mode interference (MMI) pattern of straight and four-crossing waveguide is captured using a 20X/0.45 NA objective, scale bar 25 μm, while that of the SIM chip is captured using a 60X/1.2 NA objective, scale bar 5 μm. To see the well-defined fringe patterns in case of SIM chips, the interference angle between the overlapping single mode waveguides is chosen to be 20°. The orange markers on the figures indicate the extent and orientation of the illuminating frequencies. Scale bar 500 mm^-1^ for the straight and four-arm junction waveguide and 5 μm^-1^ for the SIM chip. As shown in the figure, the illumination spatial frequencies are pre-dominant along the vertical axis for the straight waveguide geometry. To generate a more isotropic illumination scheme, four-crossing waveguide chips and SIM chips are employed. The geometry of the four-crossing waveguide generates illumination frequencies pre-dominantly along two orthogonal directions as showcased here. The SIM chips can help create a better isotropic illumination scheme, by creating interference patterns oriented at 0°, 120° and 240° at the sample plane. However, in this work we have utilized the SIM chip for 1D resolution improvement only. The Fourier peaks of the illumination pattern generated using the SIM chip along one of the axes is shown by the orange circles. The intensity bars alongside each image indicate the range of the pixel values in the images.


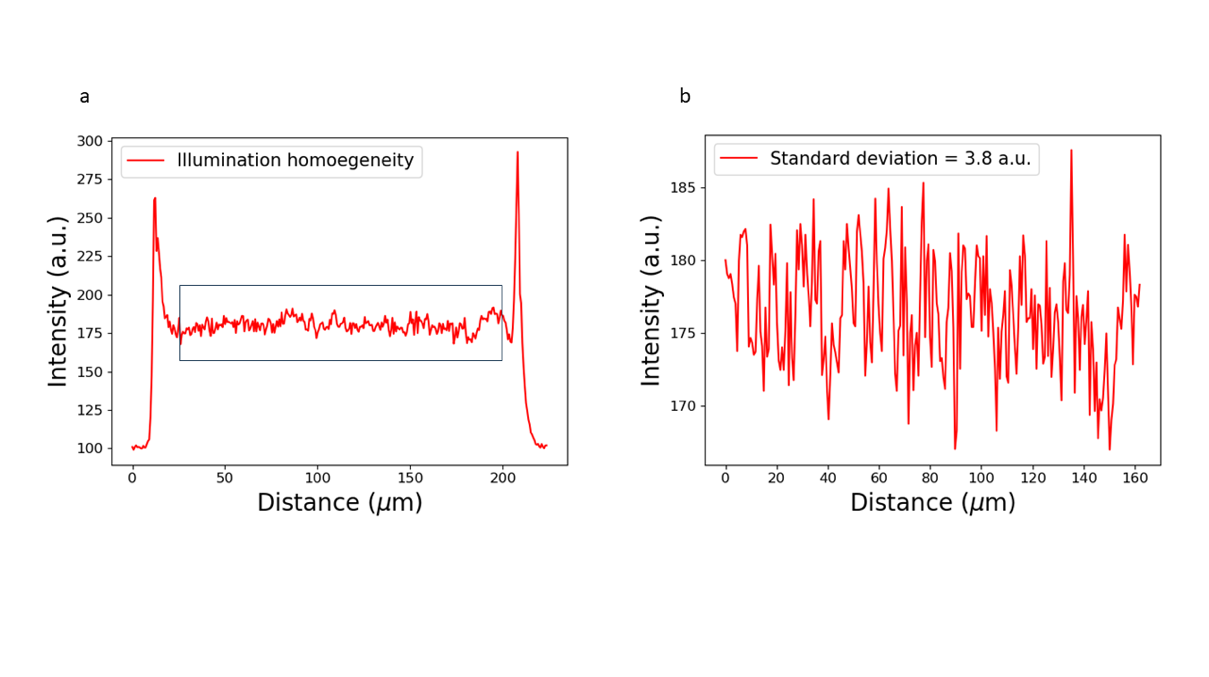


**Figure. S2b:** (a) Intensity variation along the width of a 200 µm waveguide is plotted. The two tallest peaks in the plot correspond to the edges of the waveguide. (b) The black box in (a) is zoomed in and shown for better visualization purposes. The standard deviation of intensity over approximately 160 µm is 3.8 a.u.

**Table 1:**

| **Sl. No.** | **Excitation wavelength** | **Propagation loss** |
| --- | --- | --- |
| 1 | 488 nm | ≈ 10 dB/cm |
| 2 | 561 nm | ≈ 2.5 dB/cm |
| 3 | 660 nm | ≈ 1dB/cm |

Table1: Propagation loss of a straight waveguide at different excitation wavelengths.

**3. Simulation study and experimental verification of influence of multi-mode illumination patterns for usage in IFON**

The following simulation studies elucidate how the multi-mode illumination pattern-induced correlation is mitigated to generate the super-resolved images in EPSLON.

Simulation1: The raw-image stack consists of three synthetic ring-like structures (220 nm, 260 nm and 300 nm from left to right). The data stack is then convolved with a PSF (220 nm) and down-sampled six times. Various types of noise (mixture noise with cytosol background, Poisson noise, Gaussian noise, out-of-focus light and baseline background) are then added to generate the final raw image stack. The emitters have an on/off fluctuating behavior. The fluctuation rate (On Time ζ_on_/Off Time ζ_off_) is to $\zeta_{\mathrm{on}}=1.67\times200$ frames and $\zeta_{\mathrm{off}}=2.5\times200$ frames, i.e., $\zeta_{\mathrm{on}}/\zeta_{\mathrm{off}}\approx2/3.$ The label density is set as 10000/µm^2^ and all emitters have the same intrinsic brightness. An image stack of 100 frames is then reconstructed using 2^nd^ order SOFI (SOFI^2^) and 2^nd^ order SACD (SACD^2^). The structural similarity (SSIM) score is provided alongside each of the reconstructions as well.


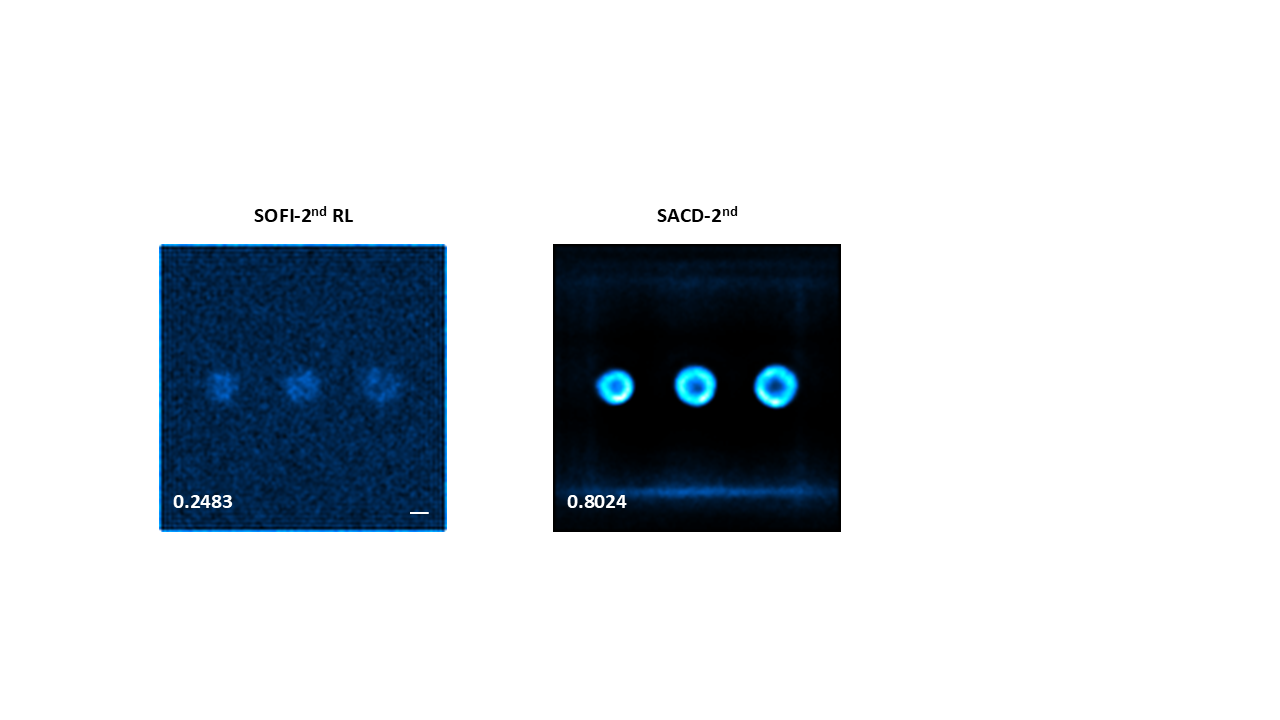


**Figure. S3**: Comparison of reconstructions using 2^nd^ order SOFI and 2^nd^ order SACD on the synthetic data set having slow intrinsic fluctuations ($\zeta_{\mathrm{on}}=1.67\times200$ frames and $\zeta_{\mathrm{off}}=2.5\times200$ frames). Scale bar 500 nm.

It is seen that SACD^2^ has been able to achieve a higher SSIM score than SOFI^2^ for the slow fluctuation rate scenario we have chosen here, Fig. 3. The higher SSIM score of SACD is attributed to the pre-deconvolution steps involved in its reconstruction pipeline. This behavior is documented in Ref. [Zhao, Weisong, et al. "Enhanced detection of fluorescence fluctuations for high-throughput super-resolution imaging." *Nature Photonics* (2023): 1-8.] where SACD^2^ is shown to have a high convergence even with just 20 frames.

Simulation2: The difference as opposed to Simulation1 is that the raw image stack is now multiplied with multi-mode illumination pattern. This will correspond to the situation where these very slowly fluctuating emitters are placed on top of the core-cladding interface of the waveguide. Therefore, these emitters get illuminated by the mode patterns of the waveguide. Then the final image at the camera plane is the product of the waveguide mode pattern and slowly fluctuating emitters convolved with the PSF (220 nm).

For simplicity, the mode patterns for the straight waveguide considered here are assumed to have a single spatial frequency and oriented only along the vertical-axis as shown in the figure below. Then each image in the image stack is multiplied with a phase-shifted fringe pattern. The phase shift is to mimic the piezo stage oscillating along the input facet of the waveguide. The phase shift of the interferogram is chosen to change by 2π radians over 10 frames. SOFI^2^ and SACD^2^ reconstructions along with the SSIM scores are given below.


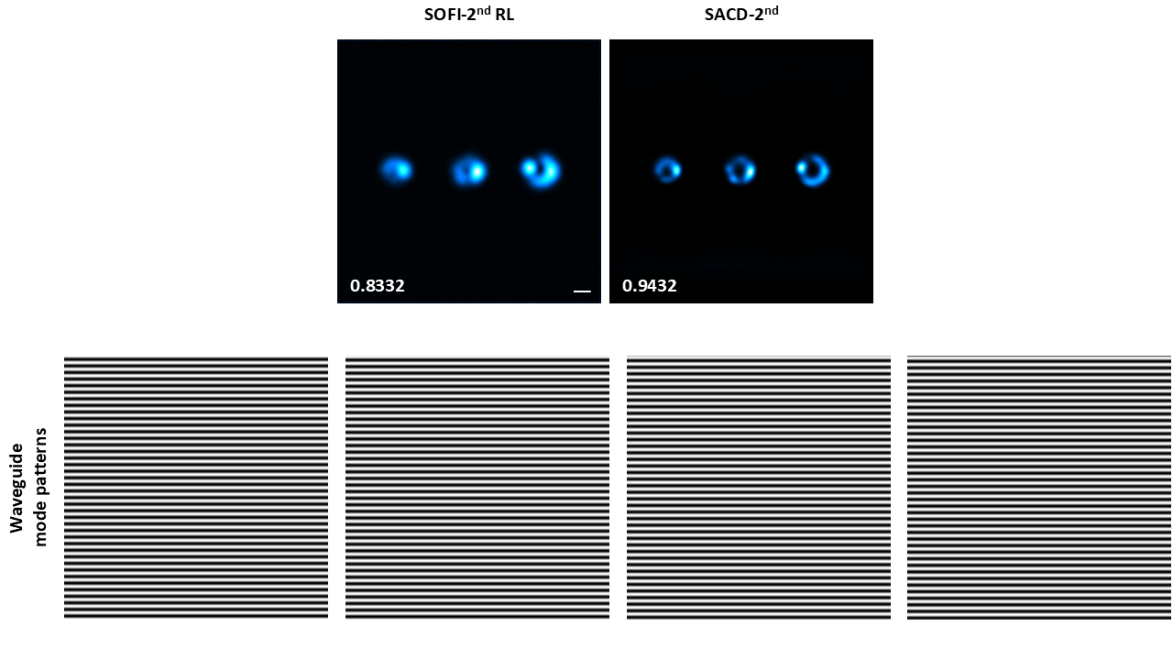


**Figure. S4**: Influence of mode patterns of a straight waveguide on reconstructions using 2^nd^ order SOFI and 2^nd^ order SACD on the synthetic data set with slow intrinsic fluctuations ($\zeta_{\mathrm{on}}=1.67\times200$ frames and $\zeta_{\mathrm{off}}=2.5\times200$ frames). A few of the phase-shifted mode patterns used for illuminating the sample is also shown. Scale bar 500 nm.

The SSIM score has improved from 0.2483 to 0.8332 for SOFI^2^ and from 0.8024 to 0.9432 for SACD^2^. This implies that the intensity-fluctuations induced by the waveguide mode patterns have helped achieve a better reconstruction. An important observation is that the non-uniform waveguide illumination induces artifacts (unresolved regions) in the image. This can be seen as bright points in the reconstructed synthetic structures in Fig. 4. These points lie along the fringes, i.e., horizontally. This arises due to the correlation between the different emitters.

Simulation3: The experimental particulars used for the emitters are the same as in the previous two cases. Here the difference arises solely because a four-arm waveguide is used to illuminate the slowly fluctuating emitters. The use of a four-arm waveguide helps mitigate the correlation between the emitters due to the random nature of the sub-diffraction sized speckle patterns. This is because these waveguides are highly multi-mode and all the four waveguides overlap in the imaging area as shown in Fig. 2b of the main text. Therefore, as the piezo stage oscillates the coupling objective along the input facet of the waveguide, a different set of modes get excited in the overlapping waveguides and thereby, induces stochasticity in the illumination pattern which helps to mitigate the illumination induced correlation.

A few of the different mode patterns used for illuminating the emitters are provided in the figure below. For simplicity, we have considered mode patterns with a single spatial frequency but with different azimuthal orientations for each frame as shown below in Fig. 5. To introduce the effect of the piezo stage oscillation along the input facet of the waveguide, these interferograms are given a phase shift of 2π radians over 10 frames. During the experiments, there will be multiple spatial frequencies and the mode patterns will be more chaotic due to the multi-mode waveguides employed.


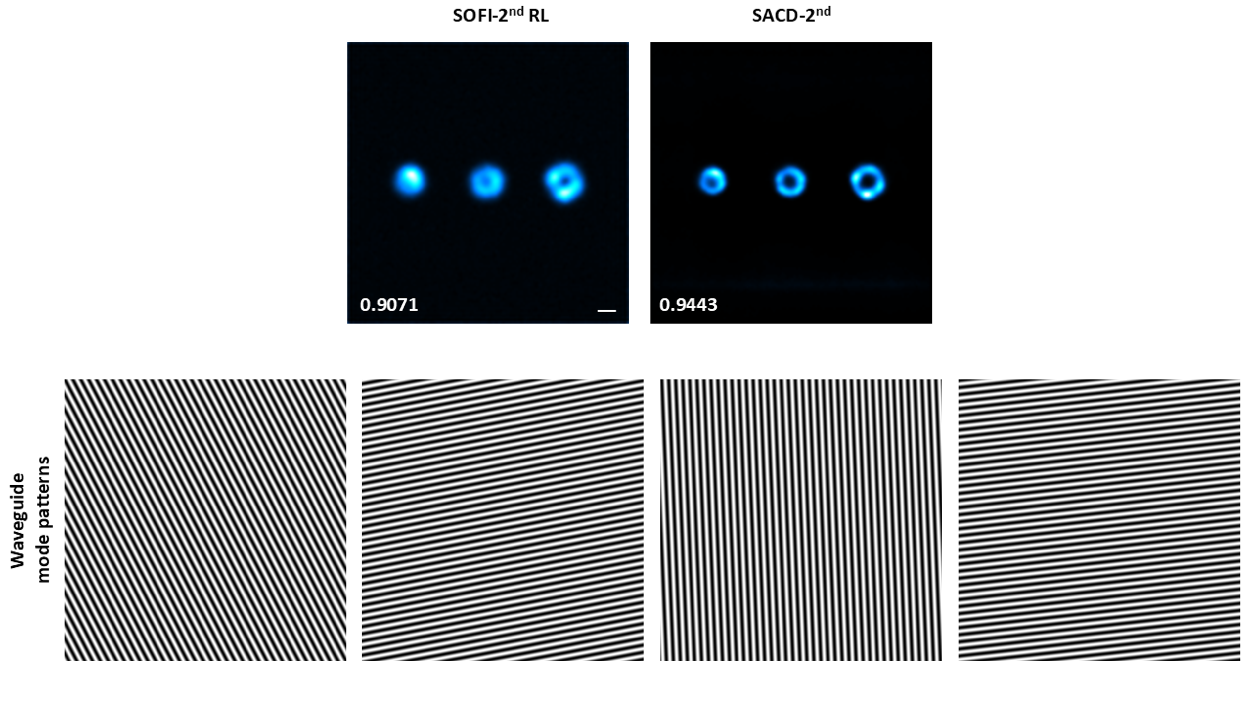


**Figure. S5**: Influence of mode patterns of a four-arm waveguide on reconstructions using 2^nd^ order SOFI and 2^nd^ order SACD on the synthetic data set with slow intrinsic fluctuations ($\zeta_{\mathrm{on}}=1.67\times200$ frames and $\zeta_{\mathrm{off}}=2.5\times200$ frames). A few of the phase-shifted mode patterns with different azimuthal orientations is also shown. Scale bar 500 nm.

The main finding is that using a four-arm waveguide, we obtained the highest SSIM score for SOFI^2^ and SACD^2^. SSIM score for SOFI^2^ is 0.9071 and for SACD^2^ is 0.9443. The artifacts (unresolved areas) have been mitigated due to reduced correlation arising due to different orientations of the illumination patterns in each frame.

Experimental validation: The simulation studies concluded that a four-arm waveguide geometry is ideal for mitigating the correlation value between the different emitters so that SOFI^2^ and SACD^2^ can generate super-resolved images. Therefore, in this experiment a four-arm waveguide geometry is employed to illuminate 195 nm polystyrene beads placed on top of its core-cladding interface. An image stack of 100 frames is captured by oscillating the coupling objective mounted on the piezo stage and is given as input to SOFI^2^ and SACD^2^. The reconstructed EPSLON images are shown and the correlation values between the emitters at 4 different regions of interest (ROI) are also provided in Fig. 6.


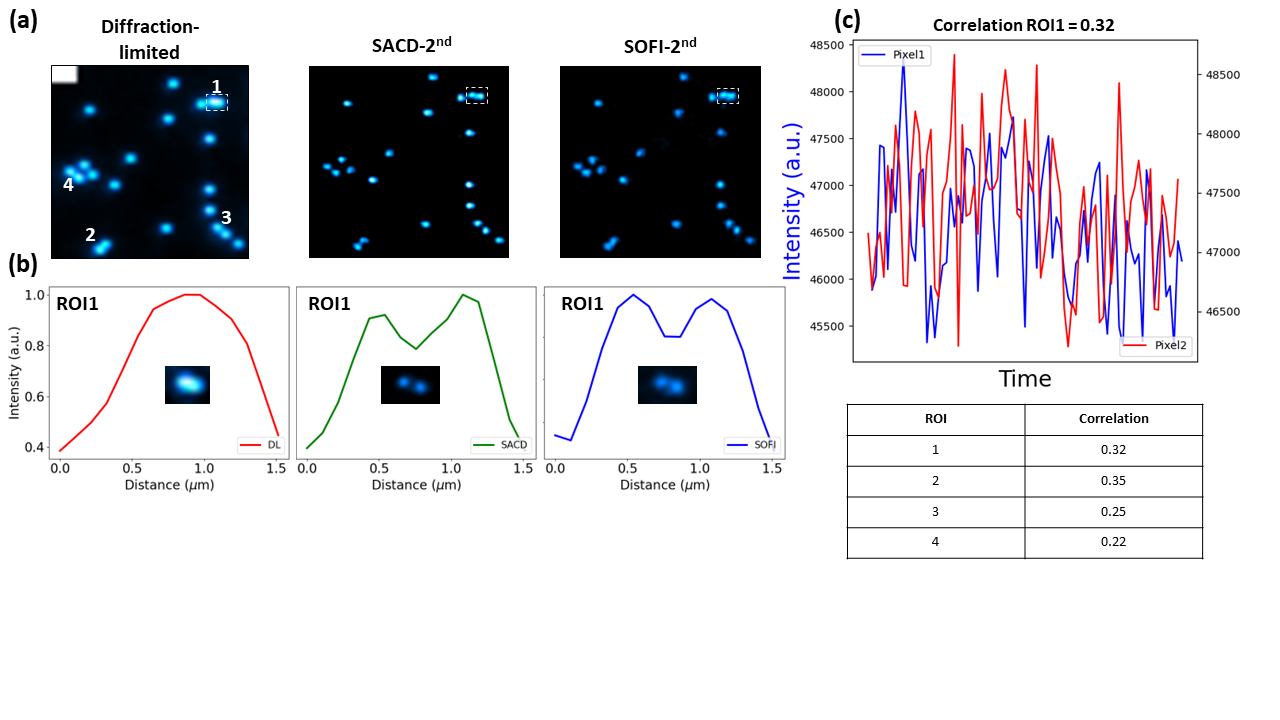


**Figure. S6**: **Experimental demonstration of low correlation value in four-arm waveguide geometry for EPSLON**. (a) Diffraction-limited (DL) image and its corresponding 2^nd^ order SACD and 2^nd^ order SOFI reconstructions are shown, scale bar 2 µm. DL image is the averaged intensity image of 100 frames as the piezo stage oscillates along the input facet of the four-arm waveguide. Four different regions of interest (ROI) labeled ‘1’, ‘2’, ‘3’ and ‘4’ are shown in the DL image. (b) The red, green and blue line plots correspond to the intensity variation (normalized) in ROI1 of DL, SACD^2^ and SOFI^2^ images. The insets in the plots provide a magnified view of ROI1 in the DL (unresolved beads) and reconstructed images (super-resolved beads). (c) Line plot shows correlation between the two adjacent pixels at the center hosting the unresolved emitters in region ‘1’ in the DL image. The correlation value is 0.32 and the correlation plot is shown. SACD^2^ and SOFI^2^ resolve the beads due to the low correlation in the DL image. (d) The correlation values in ROI2, ROI3 and ROI4 are given in the table.

Thus, in our manuscript to avoid the influence of the correlation arising due to active modulation of the PL, two strategies have been adopted:

(i) straight waveguide (Fig. 2a in main text) and SACD^2^. The high-index core material and interference between the multiple modes in straight waveguide helps generate sub-diffraction sized speckle patterns that is further employed by SACD^2^.

(ii) four-arm waveguide (Fig. 2b in main text) and SOFI^2^ or SACD^2^. The high-index core material plus the counter propagating highly multi-mode waveguides creates sub-diffraction sized speckles that vary stochastically due to the piezo scanning along the input facet of the waveguide. Such a strategy helps generate sub-diffraction sized speckle patterns that can be employed by SACD^2^, SOFI^2^ and BlindSIM.

**4. Schematic diagram of the imaging setup for EPSLON**


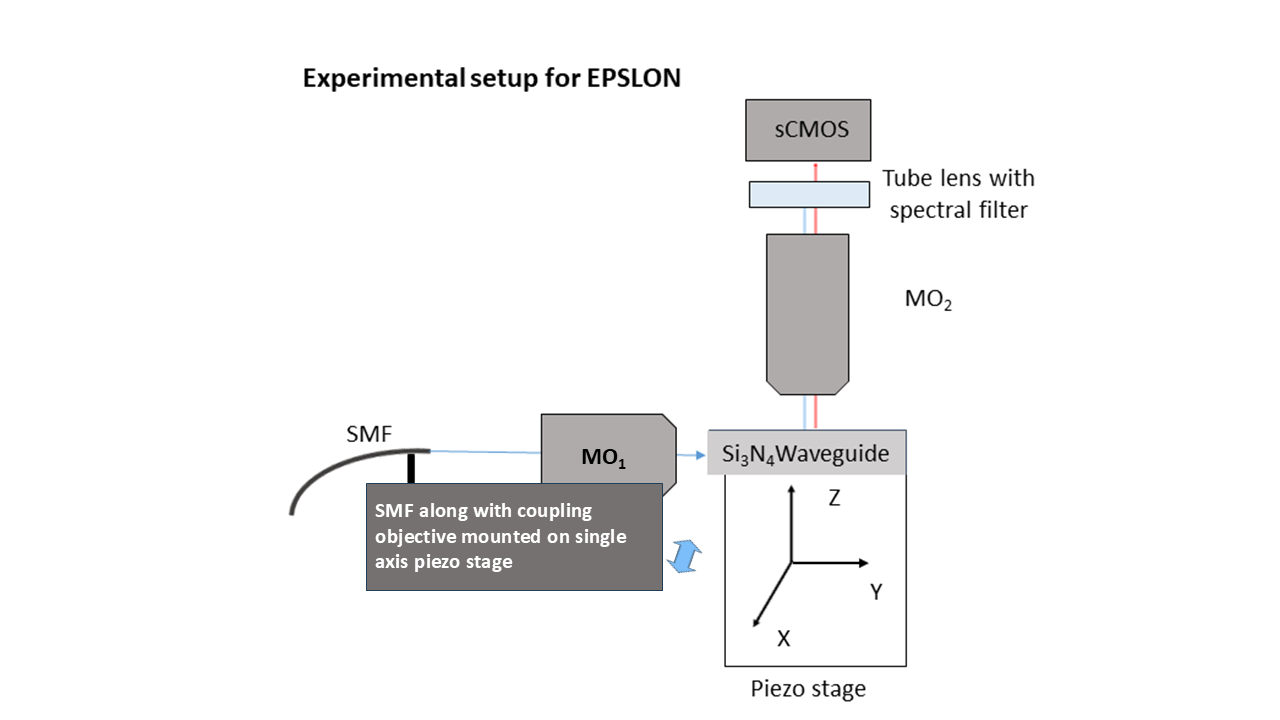


**Figure. S7. Schematic diagram of experimental setup for EPSLON**. Laser guided by a single mode fiber (SMF) held on a X-piezo stage by vacuum chuck is collimated and directed to the back-focal plane of a coupling objective (MO_1_ - Olympus LMPanFL N 50×/0.5 NA). The light is focused by MO_1_ and coupled into a Si_3_N_4_ waveguide mounted on a XYZ piezo stage. The coupled laser light in the waveguide induces a broadband photoluminescence in its core. Now, any index perturbation at the core-cladding interface of the waveguide scatters both the guided coherent, shown in blue, and the incoherent PL light, shown in red, into the far-field. Then another detection objective MO_2_ collects and directs this scattered light through a tube lens with spectral filters onto a sCMOS Hamamatsu C13440-20CU camera**.** The spectral filters are chosen to reject the coherent laser light and transmit only the incoherent PL light onto the camera. During the image acquisition, the coupling objective is oscillated along the input facet of the waveguide to excite the various modes supported by the waveguide. Typically, this scan length is chosen to span the width of the waveguide. At each scan location, various modes of the waveguide are excited with different amplitudes, a diffraction-limited (DL) image is acquired. The number of DL images acquired depends on the reconstruction algorithm employed and it is ensured that the sum of all the DL images is devoid of any non-uniformly illuminated areas at the sample plane. The captured images are then post-processed using SIM or IFON algorithms to generate the super-resolved EPSLON images.

**Investigation of the PL: Experiment to measure the ratio of transmitted to confined photoluminescence**

In the first experiment, the ratio of PL light that is confined inside the waveguide to the PL light transmitted into the far-field is quantified and found to be more than two orders of magnitude. The experimental configuration is shown below in Fig. 8 below. Laser light (488 nm) is coupled into a 400 µm wide Si_3_N_4_ waveguide using a coupling objective. This guided light will induce broadband incoherent photoluminescence (PL) inside the core. PL emitted inside the core will be both transmitted into the far-field and confined inside the core. The confined light will get guided along the length of the waveguide, attenuated by the propagation loss for that wavelength (see Table 1 above). At the output facet of the waveguide, Detection objective 1 is used to collect the guided confined light. A combination of long-pass and band-pass filter ensures that the coherent coupling laser light is blocked and only the incoherent PL guided light reaches the camera. The PL light collected by Detection Objective 1 is shown as inset in Camera which is the experimentally obtained image of the PL emanating from the output facet of the waveguide.

Now, the transmitted at the imaging area is captured by Detection Objective 2. The same combination of long-pass and band-pass filters ensure that only the transmitted incoherent PL light reaches the camera, and the coherent laser light is blocked. The inset in camera shows the multiple modes overlapping at the imaging area of the waveguide. This experiment is repeated multiple times and the counts reaching the cameras are measured. The ratio of PL transmitted (Detection objective 1) to PL confined (Detection objective 2) is approximately 0.01.


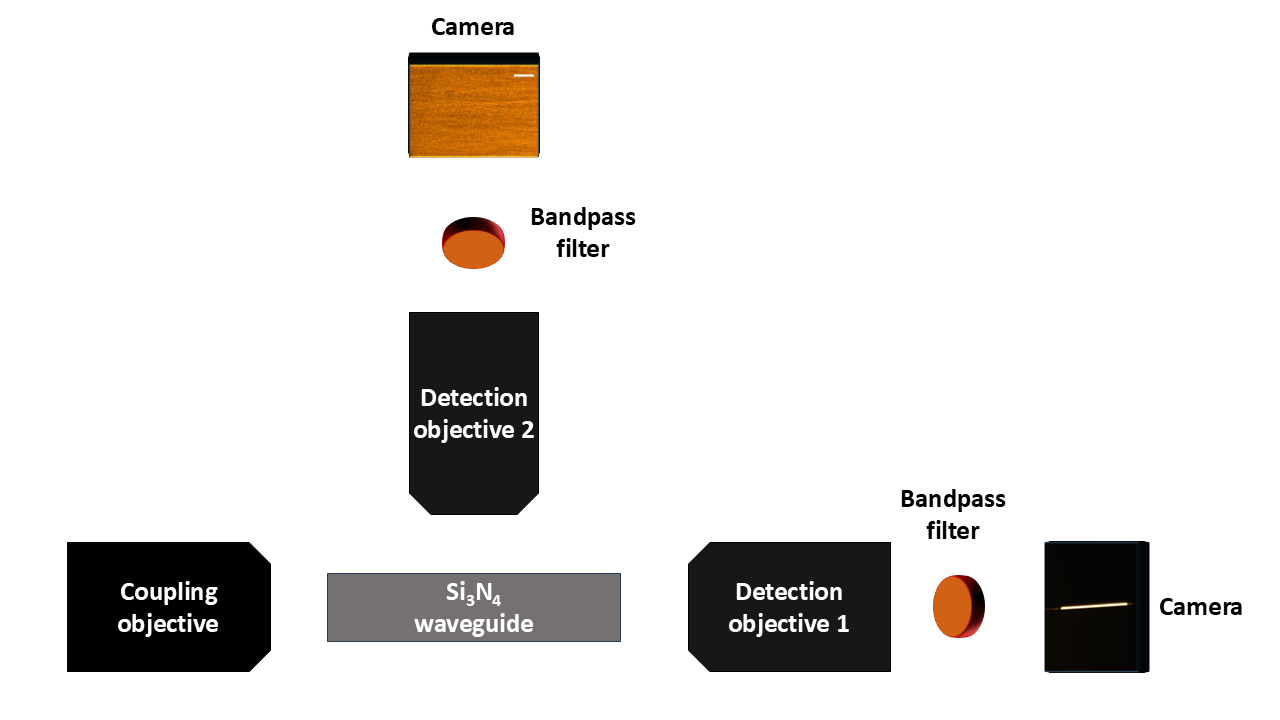


**Figure. S8a: Schematic of the experimental setup to measure the ratio of transmitted photoluminescence to the confined photoluminescence.** A coupling objective is used to guide laser (488 nm vacuum wavelength) along the length of the Si_3_N_4_ waveguide. A combination of long-pass (561 nm Semrock EdgeBasic LWP) and bandpass filter (592/43 nm EOTECH SPEC 67034) ensures that the coherent guided light is blocked and only the incoherent photoluminescence reaches the camera. Two identical detection objectives are used: Detection objective 1 for detecting the guided light along the length of the waveguide and Detection objective 2 for detecting the transmitted (unguided light) in the waveguide. Light is finally detected by identical scientific cameras. Actual images acquired in the experiment are provided as insets in the cameras, scale bar 50 μm. The ratio of light between what was collected by objective 1 and objective 2 is found to be more than two orders of magnitude.

**Investigation of the PL: Experimental verification of evanescent nature of EPSLON**

This experiment is extended using rat kidney section to validate that it is indeed the evanescently decaying PL light that contributes to the signal. For this experiment, a rat kidney section (embedded in glycerol) is placed on the waveguide. The kidney section lies on top of both Si_3_N_4_-SiO_2_ layer and in the imaging area (Si_3_N_4_ core) where no SiO_2_ layer is present. As can be seen in Fig. 8b below, only those portions of the rat kidney section which are placed directly on top of Si_3_N_4_ core-cladding interface scatter light into the camera. The epi-illumination image shows the presence of the tissue section on top of the SiO_2_ cladding layer. This experimentally proves that the evanescent field of the waveguide core contributes predominantly to image formation in EPSLON.


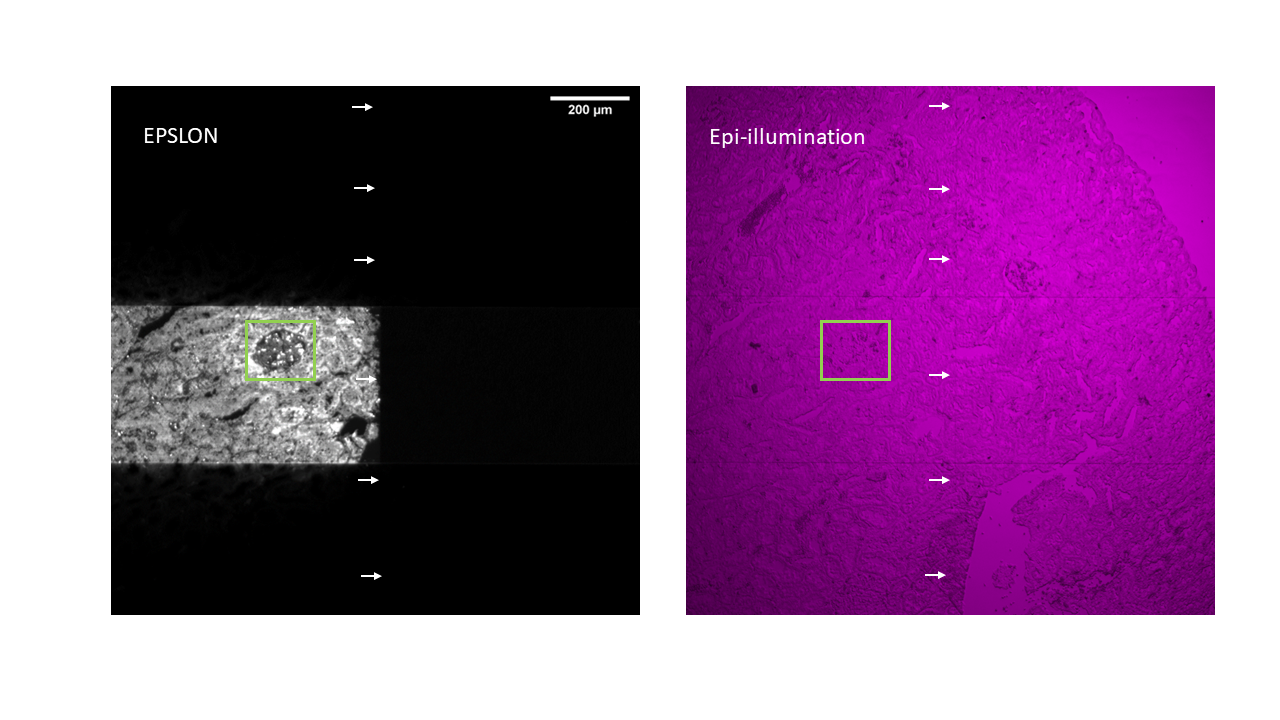


**Figure. S8b: Comparison between EPSLON and epi-illumination.** The green box simply serves as a landmark to correlate between EPSLON and epi-illumination images. The white arrows indicate the boundary where SiO_2_ cladding layer stops on the waveguide. As can be seen from the EPSLON image, signal reaches the camera from tissue sections lying directly over the Si_3_N_4_ core and no signal is obtained from tissue sections lying over the SiO_2_ cladding, scale bar 200 µm.

**5. Optical modes of the waveguide to induce intensity-fluctuations: simulation study**

Two identical square particles, 150 nm in size, are placed on top of a 10 µm rectangular waveguide. The center-to-center separation between the particles is 350 nm. The refractive index of the core is set as 2 and refractive index of cladding is set as 1. This waveguide is excited at 500 nm vacuum wavelength. The detection objective numerical aperture is set to NA = 1. Then the coherent transfer function is defined as $h_{c}=\frac{j_{1}\left( ѱ \right)}{ѱ}$, where $ѱ=\frac{2\pi r NA}{\lambda}$, where a circular aperture is assumed. Here r is the radial coordinates and λ is the vacuum wavelength of light. For simplicity, emission and detection wavelength is set to be equal, λ=500 nm. Then the spatial cut-off frequency of the system for EPSLON is defined to be twice the abovementioned coherent cut-off frequency, which corresponds to the incoherent case.

The electric field (modes of the waveguide) interacts with these particles. The scattered field is defined as the product of the electric field distribution of the guided mode and the particle. The sample space is defined as an array of zeros except at the location of the two particles, where the value is set to 1. This ensures that only the scattered fields off the two particles propagate into the far-field because in a waveguide-based illumination scheme, only the scattered fields reach the camera plane. Since identical particles are considered, phase difference between the scattered fields arises only due to different locations of the particles as mentioned in the main text. As the piezo stage oscillates the coupling objective along the input facet of the waveguide, different modes get excited with different amplitudes.


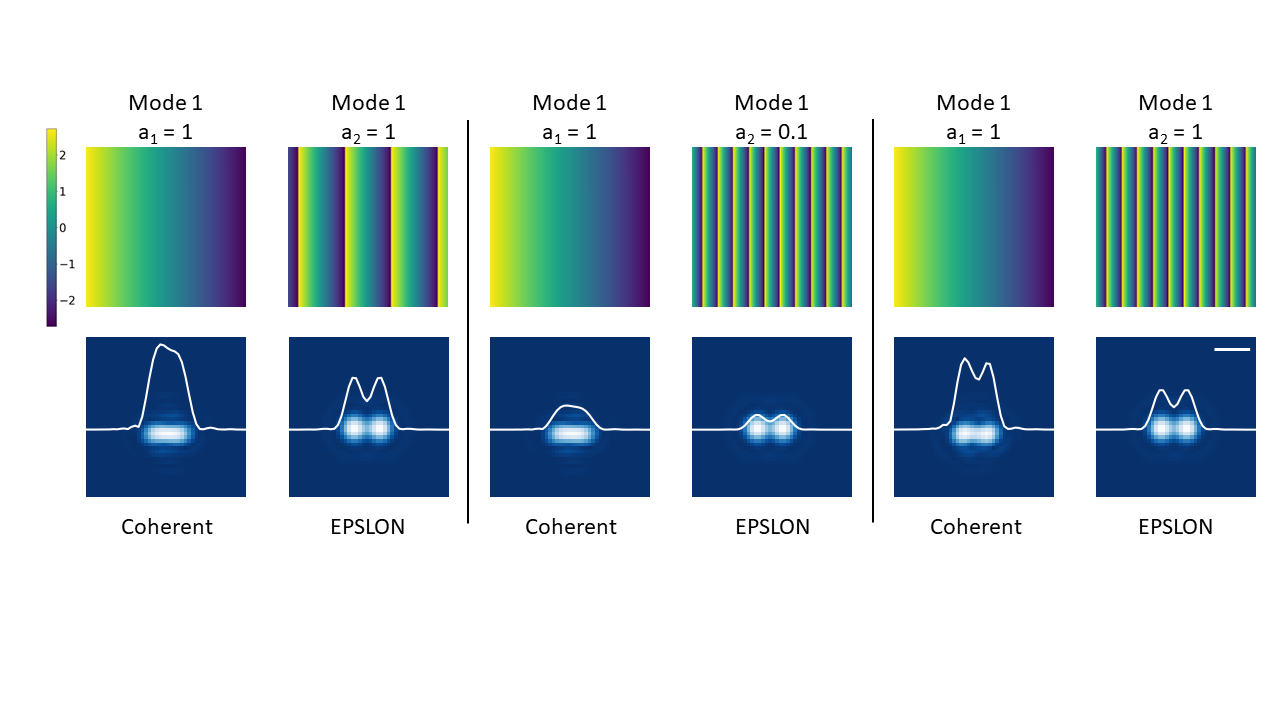


**Figure. S9: Two particle resolution comparison between waveguide-based coherent and incoherent (EPSLON) imaging.** Three separate cases are considered for comparison between coherent and incoherent EPSLON imaging. For brevity, only the fundamental mode with amplitude a_1_ and higher-order mode with amplitude a_2_ are considered interacting with the sample in each case. The colorbar provided alongside indicates the phase variation of the field across the cross-section or width of the waveguide. The sample consists of two 150 nm sized particles which are placed with a center-to-center distance of 350 nm apart on the core-cladding interface of a 10 μm Si_3_N_4_ waveguide. The particles scatter 500 nm wavelength light into a detection objective with NA = 1. The image generated at the camera plane for the coherent and EPSLON cases are shown, scale bar 500 nm. The loss of phase information in EPSLON imaging leads to similarity in images for the different excitation cases.

**6. Structured illumination microscopy using a four-arm junction waveguide**


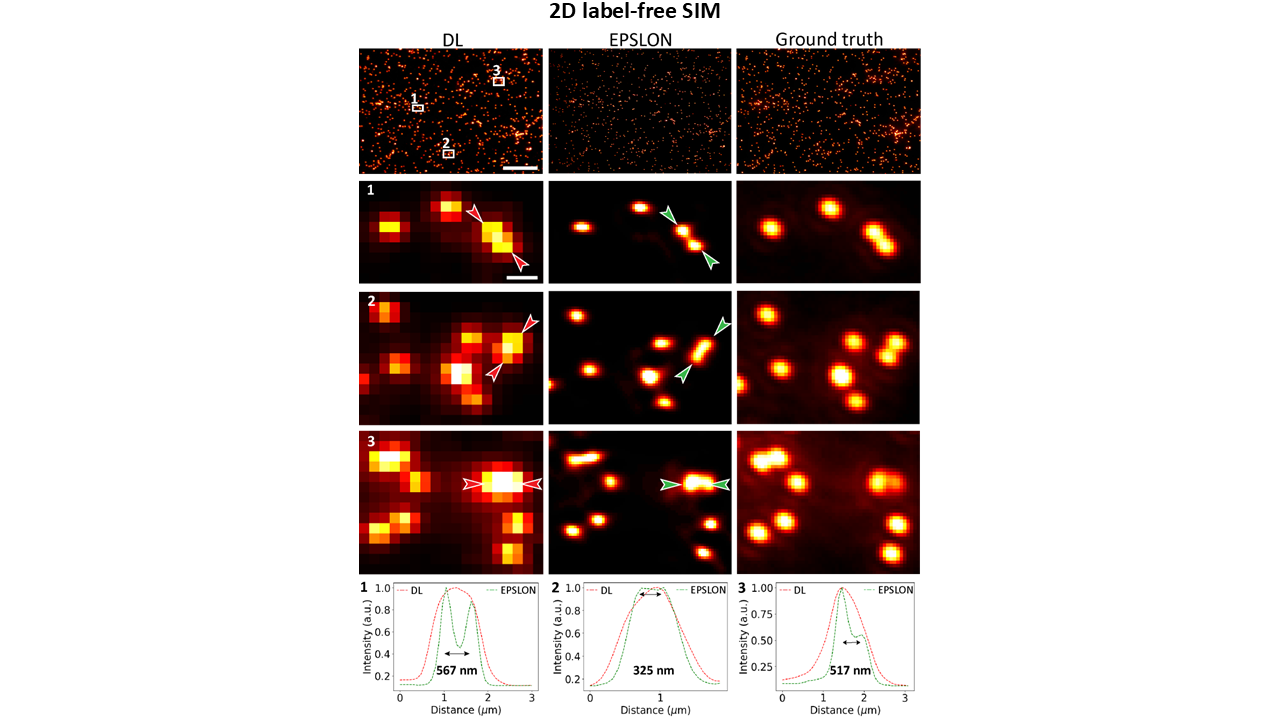


**Figure S10: EPSLON via BlindSIM for label-free 2D SIM of 195 nm polystyrene beads using four-arm junction waveguide**. Top row: large field-of-view diffraction-limited (DL), super-resolved (EPSLON) and ground-truth images are shown, scale bar 20 µm. The DL images are acquired using 0.75 NA and the ground truth optical image with a 60X/1.2 NA objective lens. Three regions of interest enclosed in white boxes in DL image are blown up and shown, scale bar 1 µm. The corresponding EPSLON and ground-truth large images are also blown up and shown alongside. The red arrowheads indicate unresolved beads in the DL images and the green arrowheads indicate the resolved beads in the EPSLON images. The line plots corresponding to normalized intensity variations along these arrows show the resolution improvement in EPSLON.

**
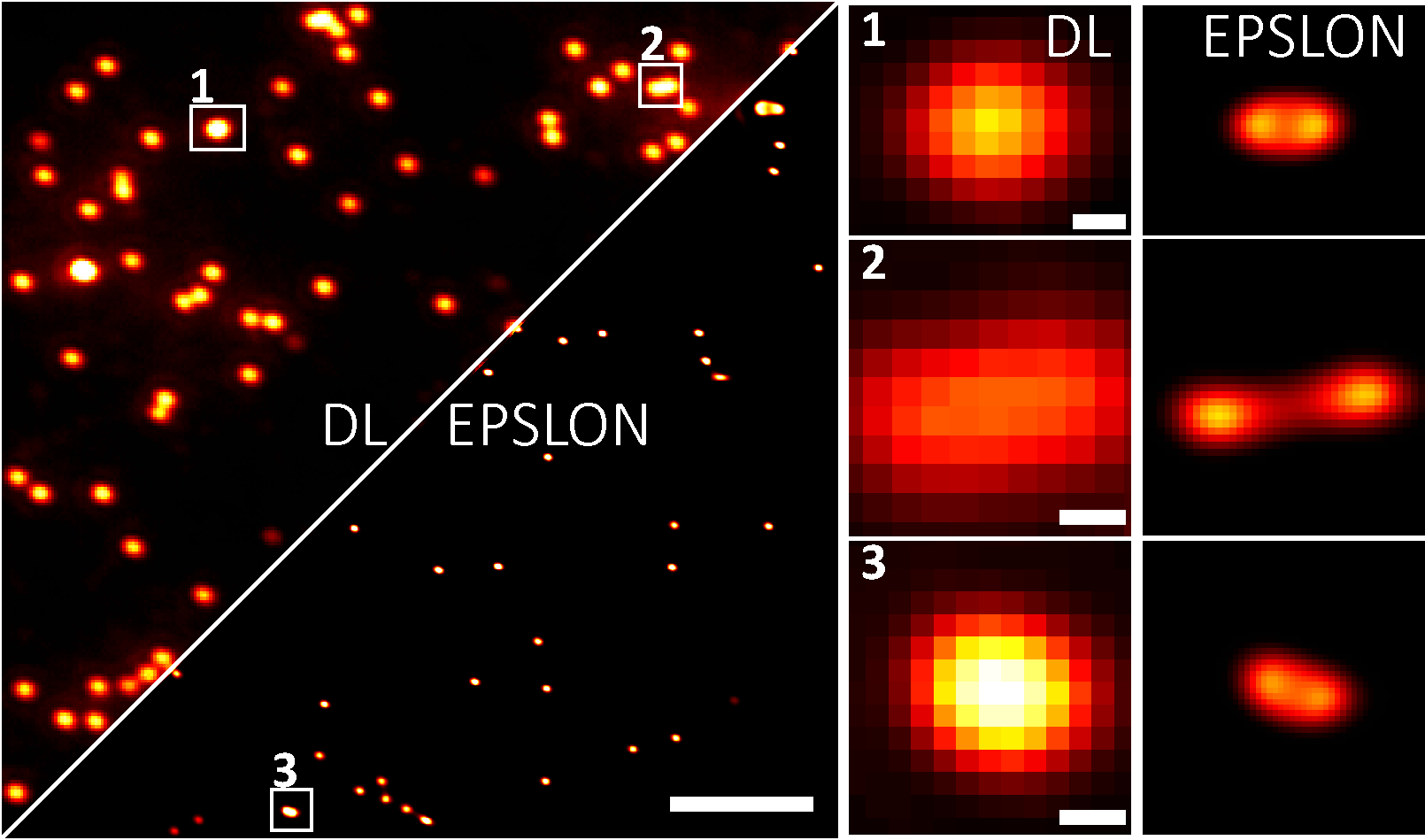
**

**Figure S11: EPSLON via BlindSIM for label-free 2D SIM of 195 nm polystyrene beads using four-arm junction waveguide** **is demonstrated using a 60X/0.9 NA objective**. Diffraction-limited (DL) and super-resolved (EPSLON) images are shown, scale bar 5 µm. Three regions of interest labelled ‘1’, ‘2’ and ‘3’ in DL and their corresponding EPSLON images are blown up and shown alongside, scale bar 125 nm.

**7. One-dimensional structured illumination microscopy using a SIM chip**

For the 1-D SIM experiment, images are acquired using a detection MO with NA = 1.2. Phase-shifted frames required for the SIM reconstruction is generated by temporarily changing the index on one of the arms of the interfering waveguides. The three phase-shifted frames are then given as input to the Fiji plugin of FairSIM. The reconstructed images and its Fourier spectra are provided in Fig. 11 below. The SIM reconstruction can clearly resolve the beads enclosed in the red inset in the DL image. The beads in the green inset in the EPSLON image are separated by 274 nm, shown in the line profile. Due to aberrations in the system, the experimental diffraction-limit of the SIM microscope is quantified to be 500 nm while the theoretical Abbe diffraction limit is $\frac{\lambda_{det}}{{2NA}_{det}}\approx272-287 nm$.


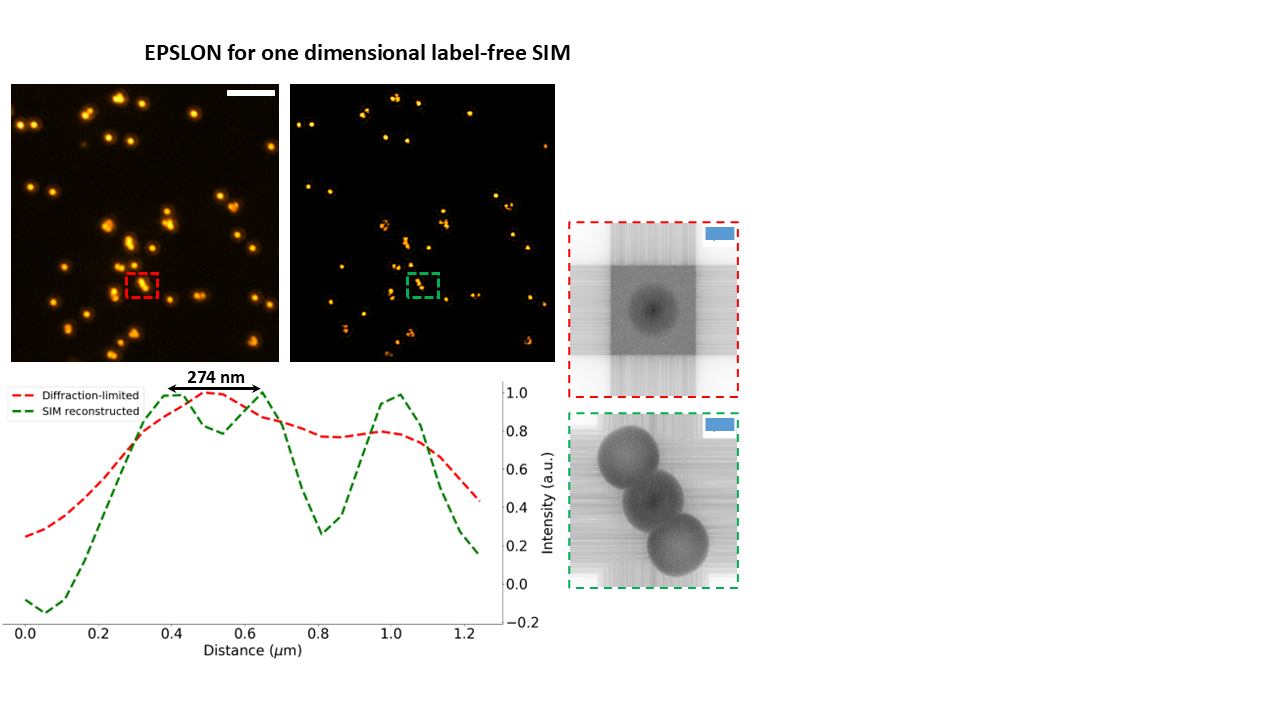


**Figure S12:** Label-free 1-D SIM of 200 nm gold nanoparticles is demonstrated, scale bar 5 μm. The line profile indicates the intensity variations in the red and green insets in the diffraction-limited DL and FairSIM reconstructed EPSLON images respectively. The line profile clearly shows the separation of two particles spaced 274 nm, which is beyond the diffraction-limit of the imaging system. The Fourier domain representation of the diffraction-limited and reconstructed image are also provided alongside, scale bar 2 μm^-1^.

**8. Investigation of the PL: Speckle size determination**

A structured illumination microscopy (SIM) waveguide chip and modes are excited at 640 nm vacuum wavelength (coupled light). The period *f* of the fringes generated is given by

$f=\frac{\lambda_{ex}}{2n_{f}\sin\frac{\theta}{2}}$

where $\lambda_{ex}$ is the excitation wavelength, $n_{f}\approx1.7$ is the refractive index of the guided mode for the Si_3_N_4_ waveguide used here and $\theta$ is the angle between the interfering waveguides.

First, we experimentally measure the fringe period generated in photoluminescence (PL) configuration. The angle between the interfering waveguides in the SIM chip is ≈25°. The period of the fringes is show in the line plot of Fig. 13 below.

Then we spin-coat the imaging area with a sub-micrometer layer of cell mask deep red fluorescent stain and excite the waveguide at the same wavelength 640 nm, i.e., acquire a TIRF image of a layer of fluorescent stain. The period of the fringes generated in fluorescence mode (TIRF) is also seen in Fig. 13 below.


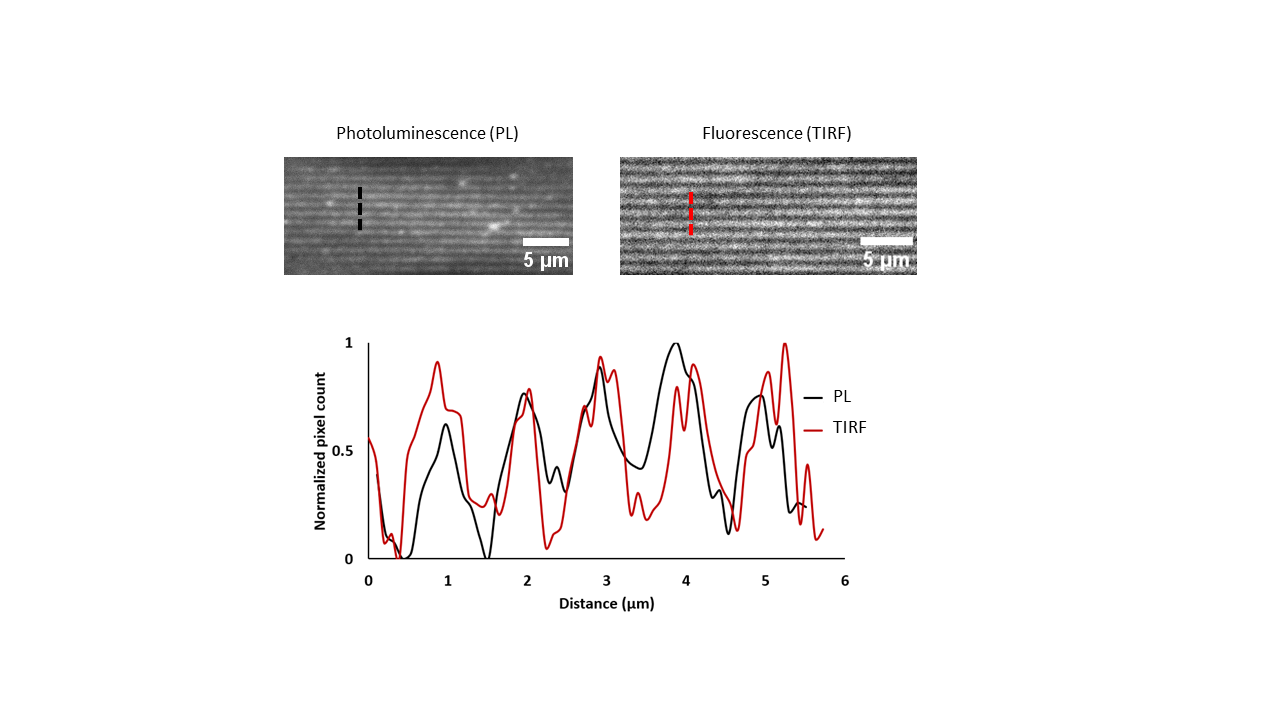


**Figure S13**: Periodicity of fringe patterns in PL and TIRF mode using a ≈25° SIM chip when $\lambda_{ex}=640 nm$. The line plots show the normalized modulation in intensity across the black and red dotted lines in the PL and TIRF images respectively. White scale bar 5 µm.

The period of the fringes (≈ 870 nm) match in both PL and fluorescence mode and closely match with the theoretical value predicted by the equation above. This implies that the period generated by the SIM chip in PL and TIRF mode is dependent only on the excitation wavelength $\lambda_{ex}$, index of the guided mode and angle between the interfering waveguides as mentioned in the formula above. The period in the SIM chips is not dependent on the emission autofluorescence wavelength $\lambda_{af}$.

Next, to show fringe period dependence on the excitation wavelength dependence$\lambda_{ex}$, the same SIM chip used above is excited in PL configuration at 640 nm and 561 nm. The periodicity and contrast of the fringe patterns are also provided alongside in Fig. 14.


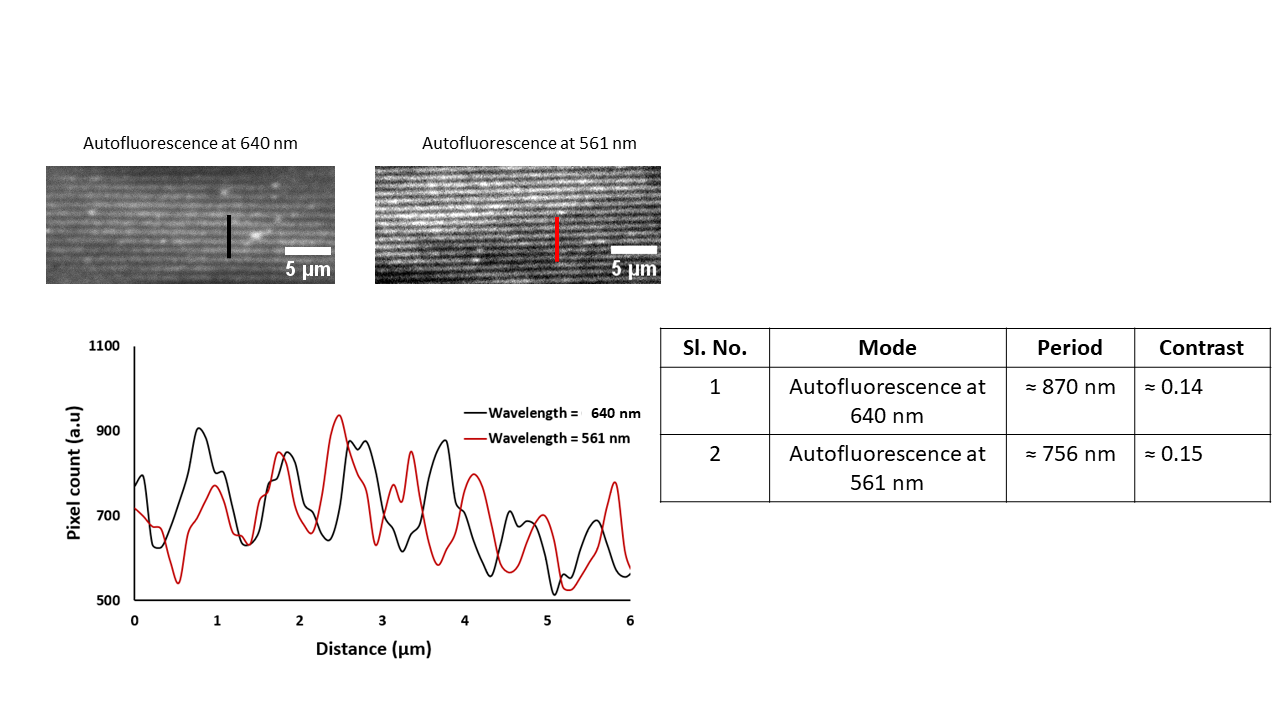


**Figure S14**: Fringe period in PL mode using a ≈25° SIM chip when $\lambda_{ex}=640 nm$ and $\lambda_{ex}=561 nm$. The line plot shows the modulation in intensity across the black and red lines in the fringe patterns. Black curve and red curve show the modulation in intensity in PL mode at $\lambda_{ex}=640 nm$ and $\lambda_{ex}=561 nm$ respectively. The table shown alongside mentions the fringe period and its corresponding contrast at $\lambda_{ex}=640 nm$ and $\lambda_{ex}=561 nm$. White scale bar 5 µm.

From Fig. 14, it is seen that the fringe period scales with $\lambda_{ex}$ as mentioned by the formula earlier. The implication of the experimental results in Fig. 12 and Fig. 13 shown above are that, for a fixed interference angle between the chips, fringe period is determined by $\lambda_{ex}$ and its corresponding effective mode index. The fringe period is ≈ 870 nm and contrast is ≈ 0.14 at $\lambda_{ex}=640 nm$. At $\lambda_{ex}=561 nm$, the fringe period is ≈ 756 nm and contrast is ≈ 0.15 and these periods closely match with the theoretical value given by the formula.

To verify that indeed we have a single spatial frequency corresponding to the excitation wavelength $\lambda_{ex}$, Fig. 14 is provided. As can be seen from the Fourier domain representation of the fringe pattern, the first order peaks, encircled within the red and green regions in Fig. 15, have a single dominant spatial frequency component. The fringe period in these SIM chips correspond to the excitation wavelength (monochromatic coupling laser light).


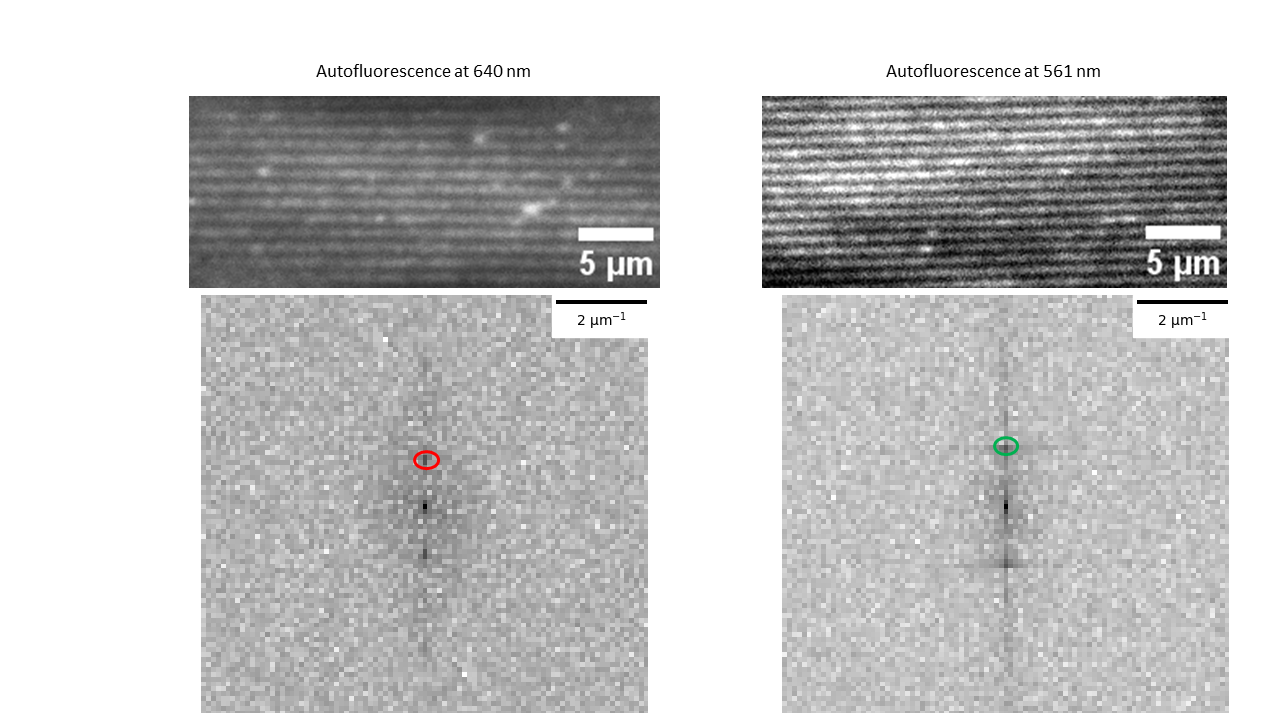


**Figure S15**: Fringe period in PL mode using a ≈25° SIM chip when $\lambda_{ex}=640 nm$ and $\lambda_{ex}=561 nm$, scale bar 5 µm. The corresponding Fourier spectrum is shown. The red and green circle indicates the first order component which corresponds to $\lambda_{ex}=640 nm$ and $\lambda_{ex}=561 nm$. Scale bar 2 µm^-1^.

Finally, to demonstrate the influence of detection wavelength $\lambda_{det}$, further experiments are carried out and the results are shown in Fig. 16. As shown in Fig. 2(e) of the main paper, the PL emission spectrum is very broad spanning more than a few hundred nanometers. Therefore, if we excite the waveguide at 488 nm, we can choose any of the filters, (FITC, TRITC, CY5), provided in Table 2 in the supplementary section. The choice of filters during experiments in this manuscript was based on maximizing the signal at the camera plane, so that the fluorescence-based super-resolution algorithms generated super-resolved images with fidelity.

To experimentally demonstrate that the influence in changing the emission filter (FITC, TRITC, CY5) is in the resolution of the final diffraction-limited image, a straight waveguide is excited at 488 nm. The scattering image and the PL images in FITC, TRITC and CY5 channels are shown alongside. As seen, the speckle patterns in all the images match well. The resolution in each channel is computed using Fourier ring correlation (FRC) and shown in the table alongside.


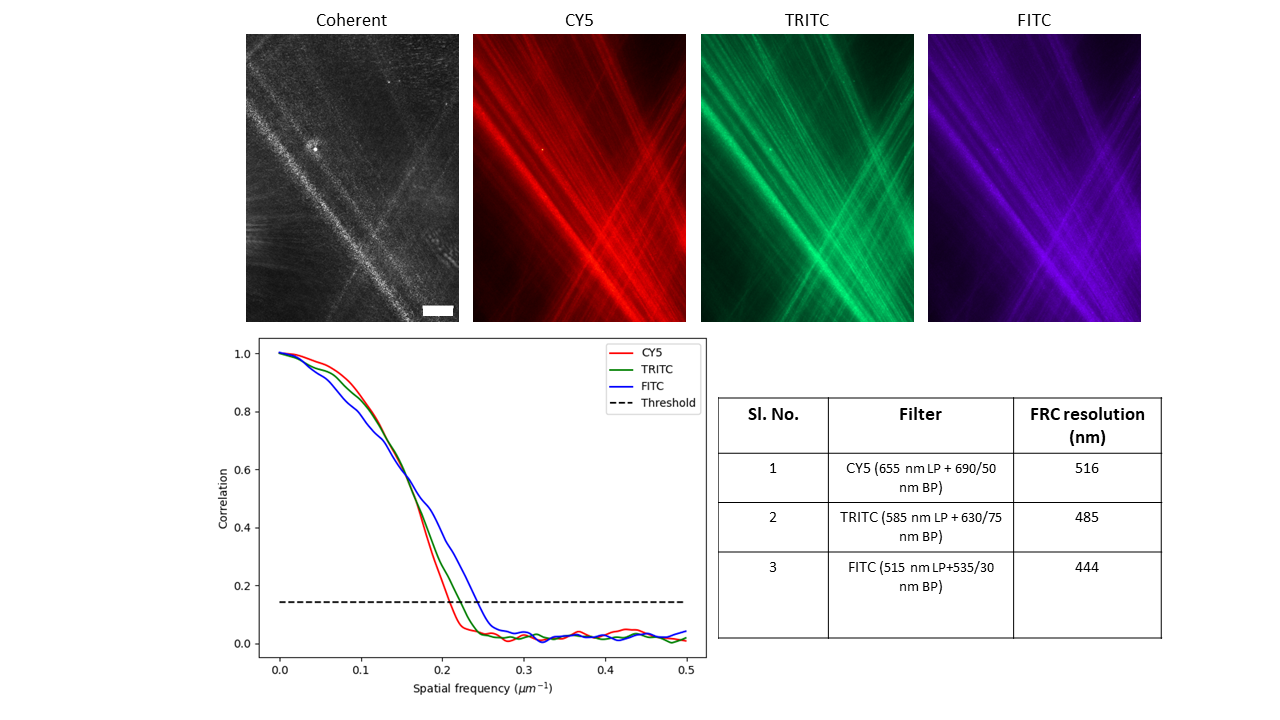


**Figure S16**: Speckle patterns, in a straight waveguide excited at 488 nm, in different filter channels and the corresponding resolution in each of the channels computed using Fourier Ring Correlation. Scale bar 25 µm.

**9. Super-resolution imaging of small EVs**

Small extracellular vesicles (EVs), are gaining attention due to their role in intercellular communication and possible clinical applications, especially for targeted drug delivery. Nevertheless, their molecular biology, as well as their therapeutic potential, is far to be completely understood. Further understanding of the spatiotemporal aspects of EVs rely on the ability to image processes such as EV secretion, uptake and biodistribution. However, imaging and tracking of small EVs has been challenging due to their small sizes (50-200 nm), and often require the use of labeling strategies, that may alter EV release and structure, prior to visualization [2]. These problems are mitigated in EPSLON: the decoupled speckle-illumination and detection paths helps visualize these structures beyond the diffraction-limit with high-contrast and without photobleaching as demonstrated in Fig. 17. The EVs used in this experiment have a size distribution of 75-250 nm, Fig. 18, and are fluorescently labeled. EPSLON and total-internal reflection fluorescence (TIRF) imaging of the same region of interest is performed. Fluorescent dyes are chosen in a way to ensure that there is no fluorescent signal reaching the camera during EPSLON imaging. 50 images are acquired in both EPSLON and TIRF mode using a detection MO with NA = 0.45. As shown in Fig. 17a and Fig. 17b, the EPSLON and TIRF images are in good agreement. The line profile in the red and green insets in Fig. 17b show a blob of light and therefore, unable to clearly resolve the EV particles. To resolve these particles, the DL image stack is given as input to the reconstruction algorithm to generate the EPSLON image. Fig. 17b shows that in the EPSLON image the EVs are clearly resolved as shown by the line profiles. To validate the EPSLON result, the same region of interest is imaged with a higher NA = 0.9 detection MO. The EPSLON matches well with the line profile of the ground truth optical image.

Small EVs were isolated from fresh urine samples collected in the morning from healthy donors. The collection of urine samples was approved by the Norwegian Regional Committees for Medical and Health Research Ethics and the participants gave informed written consent. Small EVs were isolated by differential centrifugation as previously described [3]. Briefly, urine (around 200 ml) was centrifuged at 2000×g for 15 min at room temperature (RT) to remove cells and cell debris, and then at 10,000×g for 30 min at RT to separate large particles/vesicles. The resulting supernatant was centrifuged at 100,000×g for 70 min at RT in a Ti70 fixed-angle rotor (Beckmann Coulter, IN, USA) to pellet small particles. The pellet was washed with 20 ml phosphate-buffered saline (PBS) and centrifuged again at 100,000×g for 70 min at 4°C in a Ti70 rotor. The pellet was then resuspended in 6.5 ml PBS, vortexed and centrifuged at 100,000×g for 70 min at 4°C in an MLA-80 fixed-angle rotor (Beckmann Coulter, IN, USA). The supernatant was then removed, and the pellet resuspended in 200 μl PBS (filtered through a 0.02-μm Anotop 25 filter) and stained with CellMask™ Deep Red plasma membrane dye (C10046, Invitrogen, MA, USA) according to manufacturer’s instructions. Briefly, small EVs were incubated with CellMask™ Deep Red (diluted 1:500) for 10 min at 37°C, then the unbounded dye was removed and stained EVs washed with filtered PBS using ultrafiltration devices (Amicon Ultra 0.5 mL - 3K, UFC5003234, Millipore, MA, USA) at RT. The sample was then stored at 4°C until further use. A small aliquot of the sample was used to measure the size and number of particles in the 100,000×g pellets using a Nanosight NS500 instrument (Malvern Panalytical, Malvern, UK). The sample was diluted to the optimal working concentration of the instrument (2 × 10^8^ to 1 × 10^9^ particles per ml) with filtered PBS, and then measured. Five videos of 60 sec were acquired and subsequently analyzed with the NTA 3.4 software, which identifies and tracks the center of each particle under Brownian motion to measure the average distance the particles move on a frame-by-frame basis. As shown in Fig. 18, the majority of the small EVs has a diameter between 100-175 nm (65,9% of the total) with a mode of 101 nm.

To ensure that the fluorescent signal does not contribute to the EPSLON image, TIRF imaging is initially performed at 561 nm, and the excitation light at 561 nm is kept ON until the TIRF signal gets bleached out. This along with excitation of the waveguide at 488 nm for EPSLON imaging ensured that the fluorescent and EPSLON signals do not overlap.


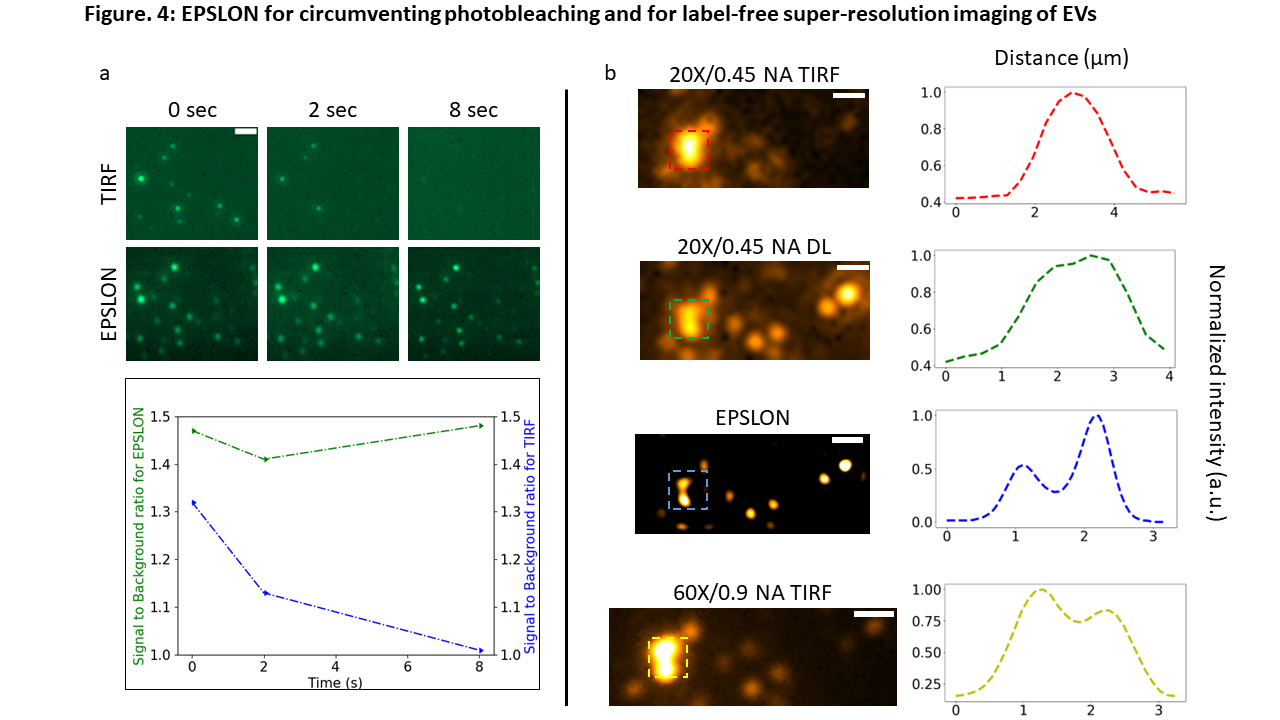


**Figure S17: EPSLON via SACD for circumventing photobleaching and for label-free super-resolution imaging of EVs.** (**a**) Time-lapse imaging comparison of extra-cellular vesicles between TIRF and EPSLON configurations. EPSLON helps to image nanosized EVs over long periods of time without photobleaching and with better signal-to-background ratio as opposed to TIRF, scale bar 5 μm. This fact is quantified in the graph where signal-to-background ratio as a function of time is plotted. (**b**) Super-resolution imaging of EVs in label-free regime using EPSLON configuration. EVs are imaged in both TIRF and EPSLON mode, scale bar 2 μm. The red, green, blue, and yellow insets correspond to EVs in diffraction-limited TIRF image, label-free diffraction-limited image termed DL, label-free super-resolved EPSLON image and TIRF ground truth image. The line profiles corresponding to each of these insets showing the intensity variation are also shown alongside. EPSLON resolves the unresolved EVs in the diffraction-limited images and this result is validated by the TIRF ground truth image acquired with a higher NA objective.

**Figure S18:** Small EVs were isolated by sequential centrifugation from healthy donor urine and their size was measured by NTA. The size distribution of small EVs is shown as percentage of particles having the indicated size normalized by the total number of particles.

**10. Rat kidney sections**

**
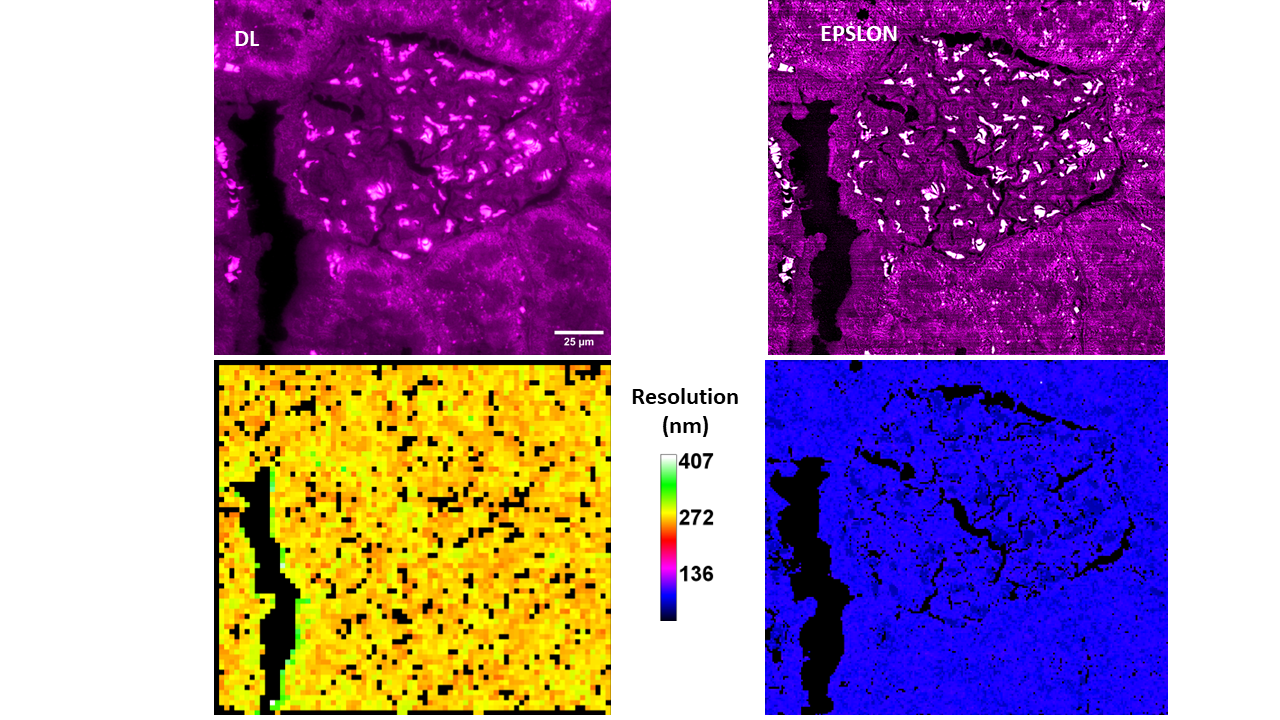
**

**Figure S19: Local FRC plot of rat kidney sections shown in Fig. 4 in main text.** Label-free diffraction-limited (DL) and its corresponding super-resolved EPSLON images are shown, scale bar 25 µm. Local FRC resolution is computed and shown below for both DL and EPSLON images. The colorbar indicates the spatial resolution in nanometers.

**12. Human kidney sections**


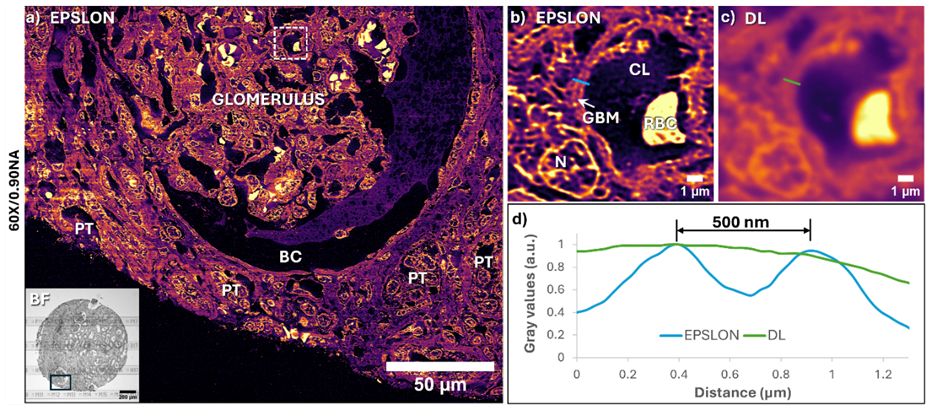


**Figure S20: EPSLON via SACD for human kidney histopathology. (a)** Lable-free super-resolved EPSLON image of a human kidney tissue section is shown, scale bar 50 µm. A mean FRC resolution of 144 nm is observed. The gray scale inset is the bright field image of the kidney section imaged with a 4X/0.10 NA detection objective, scale bar 200 µm. The pseudo-colour image illustrates the EPSLON results of the selected glomerular region in the BF image enclosed within the black box. EPSLON provides a high-contrast contextual visualization of the sample, enabling the identification of structures such as the glomerulus, the Bowman’s capsule (BC), and many adjacent proximal tubuli (PT). **(b)** The region in the EPSLON image enclosed within the white dotted box is blown-up and shown, scale bar 1µm. This figure reveals microanatomical structures including a glomerular basement membrane (GBM), a capillary lumen (CL) with a red blood cell (RBC), and the nucleus (N) of an adjacent podocyte. **(c)** The corresponding diffraction-limited (DL) view of the FOV in (b) is shown, scale bar 1µm. Note the poor contrast and resolution as compared to the EPSLON image. **(d)** Line profile measurements over the GBM reveal a separation of approximately 500 nm using EPSLON, which otherwise cannot be measured in DL modality. The blue line represents intensity variation in the EPSLON image along the region shown in (b) and green line represents intensity variation along the DL region shown in (c).

**Sample preparation**

Upon sectioning, the 8 µm thick tissue slice was scooped from a water bath and deposited on a photonic chip. The sections were dried flat at room temperature (1 × 1 h). For label-free imaging, the sample was mounted using glycerol (G5516, Sigma Life Science) and a #1.5 glass coverslips of 22 mm × 22 mm (631-0125, VWR). Subsequently, for fluorescence imaging, the coverslip was carefully removed and the tissue section was washed (3 × 5 min) with PBS to remove the glycerol. Then, the sample was incubated (1 × 15 min) in a 1:2000 solution of MitoTracker Deep Red FM (M22426, Invitrogen) in PBS. Next, the sample was rinsed (1 × 10 sec) and washed with PBS (2 × 5 min) before mounting with glycerol and covering with a #1.5 coverslip of 22 mm × 22 mm. The sample was sealed with picodent twinsil dental glue and stored at 4 °C protected from the light until imaging. The microscopy observations were performed within 1 to 3 days after labeling.

**13. Human-placenta tissue label-free imaging, preparation and characterization**

We now showcase the potential of EPSLON for the evaluation of human placental tissue sections using straight waveguides in tandem with SACD. The human placenta is a pregnancy-specific organ that plays a key role in mediating the gas and nutrient exchange between the mother and the fetus. Moreover, scientific evidence suggests a relationship between ultrastructural changes in placentas and pregnancy-related diseases such as preeclampsia [4].

Placental tissue samples are first fixed in formalin and then embedded in paraffin [5]. 4 μm sections of the tissue samples are then cut from these paraffin blocks using microtome (HM 355S Automatic Microtome, Thermo Fisher Scientific, Waltham, Massachusetts, USA). The cut sections are then placed on poly-l-lysine coated Si_3_N_4_ waveguide chips, and deparaffinized in xylene (3 × 5 min), followed by rehydration in descendent series of ethanol: 100% (2 × 10 min), 96% (2 × 10 min) and 70% (10 min).

Traditionally, the visualization of such changes was made possible through complex, costly, and slow methods such as electron microscopy. Although recent advances in fluorescence-based super-resolution microscopy have successfully enabled detailed visualizations of such ultrastructural features [6, 7], the fluorescent markers used in these imaging methods exhibit practical limitations for routine histological practice. These include (1) low stability, in the sense that the fluorescence imaging must be carried out in a relatively short period of time after sample labelling, e.g. less than a week, to avoid the issues with the decay of the fluorescent dyes; (2) special handling of the fluorescently-labeled sample, particularly to avoid light exposure that might render the dyes photobleached; and (3) extended sample preparation, in terms of cost and time, associated with the fluorescence labelling. Thus, a label-free method for achieving ultrastructural visualizations of the placental morphology would prove advantageous for the field of placental research. Here, the proposed EPSLON method alleviates these issues, enabling a fast, simple, and repeatable route for observing, for example, sub-diffraction sized placental features. We illustrate the potential of EPSLON for placental histology in Fig. 21. First, 250 optical images of a region of interest were acquired using a detection MO with NA = 1.2, and subsequently averaged to obtain a diffraction-limited image (DL), as shown in Fig. 21a. The corresponding super-resolved EPSLON image is also shown in Fig. 21a. Next, to serve as a ground truth, a scanning electron microscope image of the same sample region is performed, as illustrated in Fig. 21b. The white dotted boxes in Fig. 21a and Fig. 21b are respectively expanded in Fig. 21c and Fig. 21d. The former, Fig. 21c, reveals the enhanced contrast and super-resolution provided by EPSLON in comparison to the DL method. The latter, Fig. 21d, offers a complementary view of the same region, where the white arrows indicate features of fine correlation between EPSLON and SEM images. The mean FRC of DL image is 496 nm and of EPSLON is 176 nm,$\sim$2.8 times resolution gain. A Supplementary Movie 2 is provided to compare between EPSLON and SEM images in Fig. 21. One of the inherent advantages of photonic chip-based microscopy is large field-of-view (FOV) super-resolution imaging. Therefore, a larger FOV image of a human placenta section acquired using a 10X/0.25 NA and its corresponding EPSLON image, which is essential for histopathology applications, is shown in Supplementary Fig. 22.


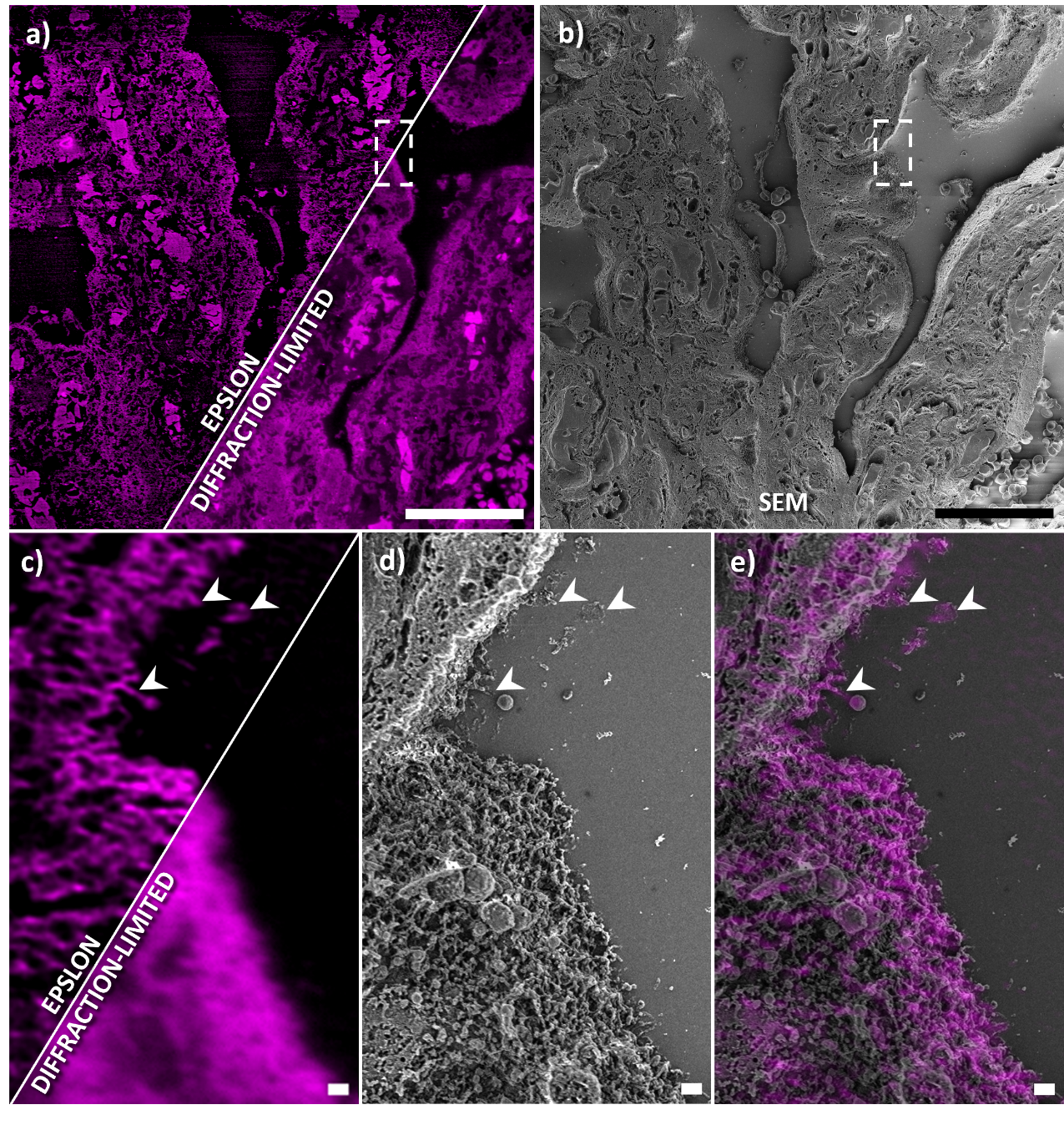


**Figure S21: EPSLON for label-free super-resolution imaging of human placenta tissue sections and benchmarking with correlative microscopy: EPLSON-SEM. (a)** A large FOV visualization of a human placental tissue section on a Si_3_N_4_ chip and imaged using a MO with detection NA = 1.2. Both DL and its corresponding super-resolved EPSLON images are shown, exhibiting a mean FRC resolution of 496 nm and 176 nm, respectively. **(b)** Same sample region acquired in a scanning electron microscope (SEM). **(c)** A zoomed-in view of the white-dotted box in (a) reveals the enhanced contrast and super-resolution provided by EPSLON in comparison to its DL counterpart. The white arrow heads denote the location of ultrastructural features with a high correlation with the SEM method. **(d)** Complementary SEM view of the same region in (c), illustrating with white arrow heads the correspondence with those seen via EPSLON. **(e)** An overlay view of (c) and (d) allows for visualizing correlation between EPSLON and SEM imaging methods. Scale bars a-b 50 µm, c-e 1 µm.

**
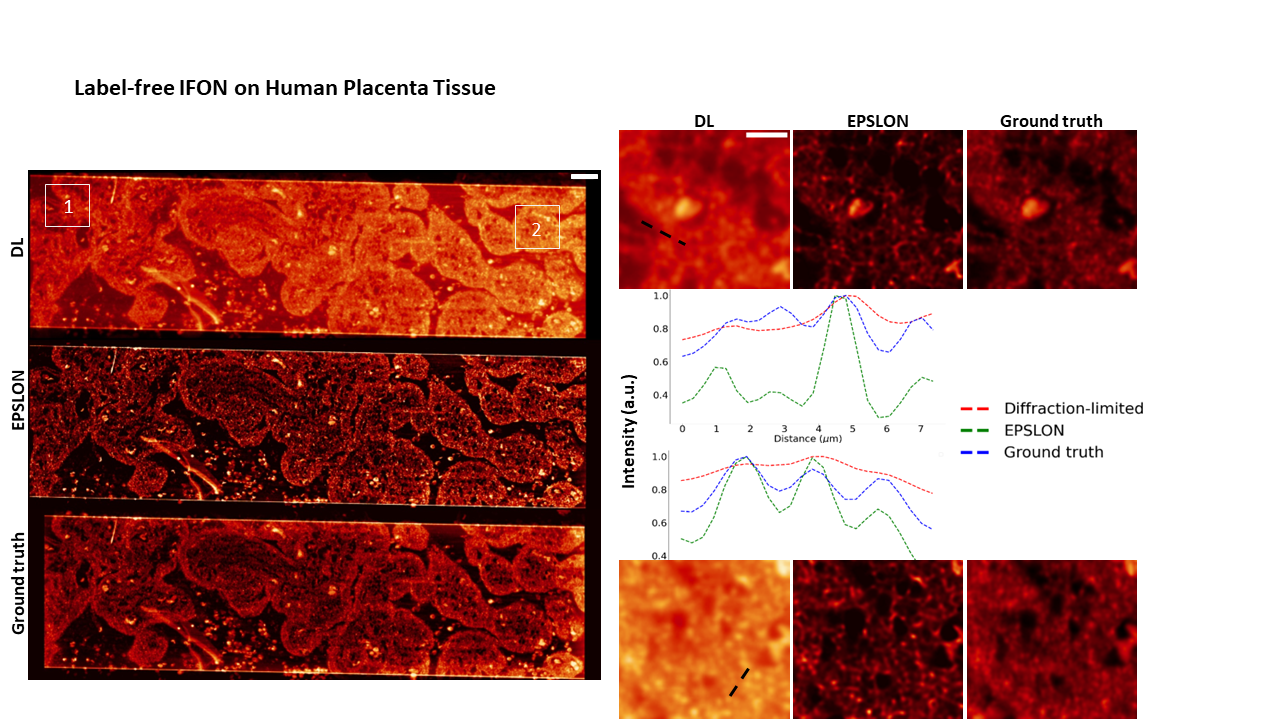
**

**Figure S22: EPSLON for label-free super-resolution imaging of human placenta tissue sections**. Large field-of-view label-free diffraction-limited image termed DL, super-resolved EPSLON and ground truth images are shown, scale bar 25 μm. Two regions marked ‘‘1’’ and ‘‘2’’ in the DL image are blown-up and shown alongside. The corresponding regions in the EPSLON and ground truth images are also magnified and shown, scale bar 10 μm. Line profiles along the white dotted lines in the magnified boxes of the DL image fail to resolve any intricate features as shown by the line plots. EPSLON images provide more details as seen in the images and they are validated by the ground truth images, which are also evidenced by the line plots.

**14. Deconvolution algorithms on diffraction-limited images acquired in EPSLON configuration**

We now compare the influence of deconvolution algorithms on the averaged diffraction-limited incoherent image. The result of various deconvolution algorithms is compared with EPSLON image shown in Fig. 3 in the main text. Post-processing the image stack via IFON helps achieve super-resolution, see Fig. 23a below where diffraction-limited (DL) and EPSLON images are shown alongside. Fig. 23b showcases the effect of deconvolving the DL image in 3a. Two-point separation of unresolved beads in the red-dotted box is seen only in EPSLON image within the green-dotted box (Fig. 23a), while such two-point separation of the beads is not observed after deconvolving the DL images, Fig. 23b.


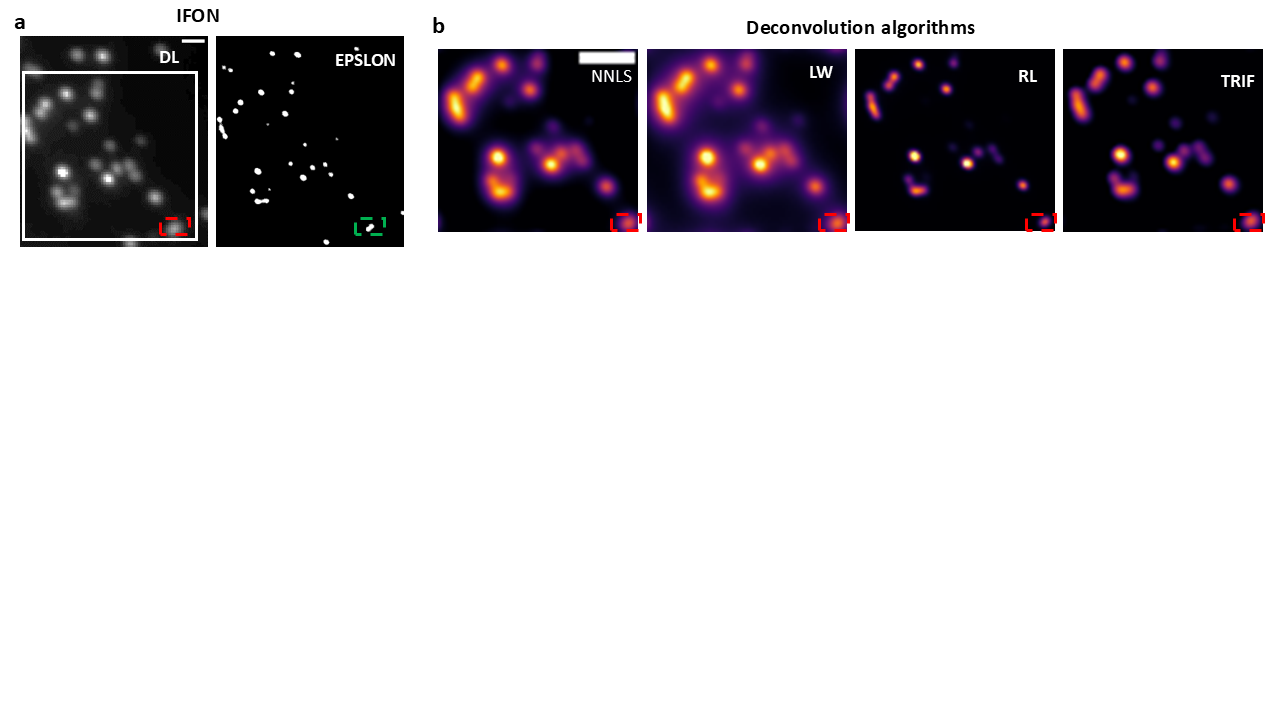


**Figure S23:** (a) Diffraction-limited (DL) image generated by averaging the image stack acquired, and its corresponding super-resolved EPSLON images are shown. The images are of 100 nm polystyrene beads deposited on straight waveguide. The red-dotted inset in the DL image shows unresolved beads, the green-dotted inset in the EPSLON image shows super-resolution. This figure is included as Fig. 3a in the main text of the revised manuscript. Scale bar 1 μm. (b) A smaller field-of-view enclosed by the white box in the DL image is then deconvolved. Various deconvolution algorithms like Non-linear least square (NNLS), Landweber (LW), Richardson-Lucy (RL) and Tikhonov Regularization Inverse Filter (TRIF) are employed. The red insets in the deconvolved images show the unresolved beads enclosed in the red dotted box in the DL image. Scale bar 2 μm. Fiji plugin is used to process the images [8].

**15. Experimental details**

The following spectral filters are used in this work:

**Table 2:**

| **Sl. No.** | **Filter Name** | **Spectral range**  **(LP: LongPass, BP: BandPass)** |
| --- | --- | --- |
| 1 | FITC | 515 nm LP + 535\30 nm BP |
| 2 | TRITC | 585 nm LP \ 630\75 nm BP |
| 3 | CY5 | 655 nm LP \ 690\50 nm BP |

**Table 3:**

| **Figure #** | **Sample** | **λ_ill_** | **Spectral filter \ MO_2_** | **Exposure** | **Reconstruction**  **Algorithm** | **# Images** | **Comments** |
| --- | --- | --- | --- | --- | --- | --- | --- |
|  |  |  |  |  |  |  |  |
| 3a | 100 nm Polystyrene beads | 405 nm | FITC \ 0.9 NA | 100 ms | SACD [9] | 100 | Order 2 used for reconstruction |
| 4 | Rat kidney sections | 488 nm | FITC\ 1.42 NA | 100 ms | SACD | 250 | Order 2 used for reconstruction. |
| 5 | Human kidney section | 488 nm | FITC/0.9 NA | 100 ms | SACD | 250 | Order 2 used for reconstruction |
| Supplementary Fig. 10 | 195 nm polystyrene beads | 561 nm | TRITC \ 0.75 NA | 100 ms | BlindSIM[10] | 50 |  |
| Supplementary Fig. 11 | 195 nm polystyrene beads | 561 nm | TRITC \ 0.9 NA | 100 ms | BlindSIM | 50 |  |
| Supplementary Fig. 12 | 200 nm gold nanoparticles | 640 nm | CY5\ 1.2 NA | 100 ms | FairSIM[11] | 3 |  |
| Supplementary Fig. 17 | (75 –250) nm Extracellular vesicles | 488 nm for PL  561 nm for TIRF | TRITC \ 0.45 NA  TRITC\0.45 NA and 0.9NA | 1 sec  100 ms | SACD | 50 | Order 2 used for reconstruction |
| Supplementary Fig. 20 | Human kidney section | 488 nm | FITC/0.9 NA | 100 ms | SACD | 250 | Order 2 used for reconstruction |
| Supplementary Fig. 21 | Human placenta tissue | 488 nm | TRITC \ 1.2 NA | 100 ms | SACD | 250 | Order 2 used for reconstruction |
| Supplementary Fig. 22 | Human placenta tissue | 488 nm | TRITC\ 0.25 NA (PL image) and 0.45 NA (Ground truth) | 50 ms | SACD | 20 | Order 2 used for reconstruction. Each image is acquired for 50 ms and 20 images are averaged to create (50 ms*20 = 1sec) to create one input image for SACD. |
|  |  |  |  |  |  |  |  |

**16. References**

1. Prieto, F., Sepúlveda, B., Calle, A., Llobera, A., Domínguez, C., Abad, A., Montoya, A. and Lechuga, L.M., 2003. An integrated optical interferometric nanodevice based on silicon technology for biosensor applications. *Nanotechnology*, *14*(8), p.907.
2. Chuo, S.T.Y., Chien, J.C.Y. and Lai, C.P.K., 2018. Imaging extracellular vesicles: current and emerging methods. *Journal of biomedical science*, *25*(1), pp.1-10.
3. Ramirez-Garrastacho, M., Berge, V., Linē, A. and Llorente, A., 2022. Potential of miRNAs in urinary extracellular vesicles for management of active surveillance in prostate cancer patients. *British journal of cancer*, *126*(3), pp.492-501.
4. Redman, C.W.G., Tannetta, D.S., Dragovic, R.A., Gardiner, C., Southcombe, J.H., Collett, G.P. and Sargent, I.L., 2012. Does size matter? Placental debris and the pathophysiology of pre-eclampsia. *Placenta*, *33*, pp.S48-S54.
5. Slaoui, M. and Fiette, L., 2011. Histopathology procedures: from tissue sampling to histopathological evaluation. In *Drug safety evaluation* (pp. 69-82). Humana Press.
6. Villegas-Hernández, L.E., Dubey, V., Nystad, M., Tinguely, J.C., Coucheron, D.A., Dullo, F.T., Priyadarshi, A., Acuña, S., Ahmad, A., Mateos, J.M. and Barmettler, G., 2022. Chip-based multimodal super-resolution microscopy for histological investigations of cryopreserved tissue sections. *Light: Science & Applications*, *11*(1), pp.1-17.
7. Villegas-Hernández, L.E., Nystad, M., Ströhl, F., Basnet, P., Acharya, G. and Ahluwalia, B.S., 2020. Visualizing ultrastructural details of placental tissue with super-resolution structured illumination microscopy. *Placenta*, *97*, pp.42-45.
8. Sage, D., Donati, L., Soulez, F., Fortun, D., Schmit, G., Seitz, A., ... & Unser, M. (2017). DeconvolutionLab2: An open-source software for deconvolution microscopy. *Methods*, *115*, 28-41.
9. Zhao, Weisong, et al. "Enhanced detection of fluorescence fluctuations for high-throughput super-resolution imaging." *Nature Photonics* (2023): 1-8.
10. Mudry, Emeric, et al. "Structured illumination microscopy using unknown speckle patterns." *Nature Photonics* 6.5 (2012): 312-315.
11. Müller, M., Mönkemöller, V., Hennig, S., Hübner, W. and Huser, T., 2016. Open-source image reconstruction of super-resolution structured illumination microscopy data in ImageJ. *Nature communications*, *7*(1), pp.1-6.
